# Galileo Broadcast Ephemeris and Clock Errors Analysis: 1 January 2017 to 31 July 2020

**DOI:** 10.3390/s20236832

**Published:** 2020-11-29

**Authors:** María Teresa Alonso, Jaume Sanz, José Miguel Juan, Adrià Rovira García, Guillermo González Casado

**Affiliations:** Research Group of Astronomy and Geomatics (gAGE), Universitat Politècnica de Catalunya (UPC), 08034 Barcelona, Spain; jaume.sanz@upc.edu (J.S.); jose.miguel.juan@upc.edu (J.M.J.); adria.rovira@upc.edu (A.R.G.); guillermo.gonzalez@upc.edu (G.G.C.)

**Keywords:** GNSS, Galileo, GPS, orbits and clock, data cleansing, anomaly detection and verification, SISA, observed satellite fault probabilities

## Abstract

A preliminary analysis of Galileo F/NAV broadcast Clock and Ephemeris is performed in this paper with 43 months of data. Using consolidated Galileo Receiver Independent Exchange (RINEX) navigation files, automated navigation data monitoring is applied from 1 January 2017 to 31 July 2020 to detect and verify potential faults in the satellite broadcast navigation data. Based on these observation results, the Galileo Signal-in-Space is assessed, and the probability of satellite failure is estimated. The Galileo nominal ranging accuracy is also characterized. Results for GPS satellites are included in the paper to compare Galileo performances with a consolidated constellation. Although this study is limited by the short observation period available, the analysis over the last three-year window shows promising results with $P_{sat}$= 3.2 × 10^−6^/sat, which is below the value of 1 × 10^−5^ stated by the Galileo commitments.

## 1. Introduction

Galileo is the European Global Navigation Satellite System (GNSS). Similar to the other GNSSs (GPS, GLONASS, and BeiDou), it provides positioning, navigation, and timing services for worldwide users.

The first phase of Galileo deployment started with a reduced constellation of four operational In-Orbit Validation (IOV) satellites launched in 2011 and 2012. After the successful completion of this initial phase, the Galileo program is currently moving toward Full Operational Capability (FOC). The first pair of Galileo FOC satellites, E201 and E202, was launched in August 2014. Unfortunately, due to an orbit injection anomaly, these satellites were placed into erroneous eccentric orbits. After these two eccentric satellites, 20 FOC satellites were successfully launched between 2015 and 2018. Currently, the Galileo constellation comprises 26 satellites, 4 IOV, and 22 FOC, transmitting on five frequencies, i.e., E1, E5a, E5b, E5, and E6 [1].

Galileo Initial Service Open Service (IS OS) was declared by the European Commission on 15 December 2016. After the reprofiling of Galileo Safety-of-Life (SoL) in the early 2010s, Galileo is meant to support augmentation for SoL services through a Dual-Frequency Multiconstellation (DFMC) Satellite-Based Augmentation System (SBAS) and Advanced Receiver Autonomous Integrity Monitoring (ARAIM) [2,3]. Integrity denotes the measure of trust that can be placed in the information supplied by the navigation system.

The characterization of Clock and Ephemeris error of the GNSSs is a key element to validate the assumptions for the integrity analysis of GNSS SoL augmentation systems. Specifically, the performance metrics of SoL applications require the characterization of the nominal User Range Errors (UREs) as well as the knowledge of the probability of a satellite or a constellation fault ($P_{sat},\;P_{const}$), i.e., when one or more satellites are not in the nominal mode [4].

The Signal-in-Space (SIS) User Range Error (URE) is typically characterized by a zero-mean Gaussian distribution with a standard deviation represented by the User Range Accuracy (URA). The GPS broadcast URA provides a conservative estimate of the user range error in the associated navigation data. Galileo satellites broadcast the Signal-in-Space Accuracy (SISA) index, but as provided today in Galileo SIS, it is not equivalent to the GPS URA. An evolution of the SISA algorithm is resultantly being developed to compute a Galileo URA [5]. The Galileo SISA is expected to be equivalent to the GPS URA as both are operated jointly in the ARAIM.

The probability $P_{sat,i}$ describes independent satellite faults (Narrow Faults) in such a way that the probability of having two satellites affected simultaneously by such independent fault modes is not greater than $P_{sat,i}\cdot P_{sat,j}$ [4]. These are faults that may occur on board one satellite and have no effect on the other satellites. On the other hand, $P_{const}$ is the probability of Wide Faults, which is when two or more satellites are in fault mode due to a common cause, e.g., due to control segment or design errors [6].

The EU-US GNSS Working Group C provided guidelines on how the GNSS Constellation Service Providers (CSP) commitment should be specified [7]. In short, the information broadcast by the CSP specified that the SIS ranging error is bounded by a normal distribution with a near-zero mean and standard deviation of less than or equal to $\sigma_{\mathrm{URA}}$ during fault-free operations.

According to the Global Positioning System Standard Positioning Service Performance Standard (GPS SPS PS) [8], a satellite is considered to be faulty (major service failure) when the Line-of-Sight (LoS) projected error is greater than a Not-to-Exceed (NTE) threshold. This NTE is defined as NTE = 4.42 × IAURA, where IAURA stands for Integrity Assured URA, which is equal to the upper bound on the $\sigma_{\mathrm{URA}}$ value, corresponding to the URA index broadcast by the GPS satellites. Indeed, the commitments of [8] state an upper bound of 1 × 10^−5^/sat/h probability of satellite fault, per satellite, per hour (which corresponds to a *k*-factor of 4.42 when considering a Gaussian distribution), and that major service faults will be flagged or removed with an average alarm delay of one hour (Mean Fault Duration) and a worst case of six hours. This implies an extreme upper bound $P_{sat}$ ≤ 6 × 10^−5^/sat of probability that at any given time a satellite observation is faulty.

In the case of Galileo, the European GNSS (Galileo) Open Service Service Definition Document (OS SDD) [9] establishes that the expected value of the Galileo probability of Signal-in-Space (SIS) fault for future configurations of Galileo during the Full Operational Capability (FOC) service provision is $P_{sat}$ = 6 × 10^−5^/sat, with NTE = 40 m. More recently, the Galileo program established a dedicated process involving the main actors, the European Commission (EC), European Space Agency (ESA), and European GNSS Agency (GSA), which is analyzing the Galileo performance to support the definition of the ARAIM concept and relative standards. The conclusions presented in the International Civil Aviation Organization (ICAO) Navigation Systems Panel (NSP) on April 2020 proposed a Galileo URA value that shall not exceed 6 m with $P_{sat}$ lower than 3 × 10^−5^/sat, which leads to NTE = 4.17 × 6 = 25.04 m (*k* = 4.17 being the factor corresponding to the 3 × 10^−5^ probability for the Gaussian distribution). Moreover, it is considered with a probability $P_{const}$ lower than 1 × 10^−4^ that, at any given time, an observed subset of two or more satellites of Galileo constellation is faulty due to the same root cause [10].

The present work aims to characterize the performance of broadcast navigation data during the first years of initial Galileo IS OS.

Previous similar studies have been conducted for GPS in [11,12]. In this paper, we apply the methodology developed in such works to Galileo satellites, taking into account the specifications of the Galileo system.

The general approach to perform such studies consists of identifying potentially broadcast erroneous navigation data by comparing “consolidated” Receiver Independent Exchange (RINEX) format [13] broadcast navigation files with precise orbit and clock reference products that are considered the truth. Potential anomalies are then verified using measurements collected by a network of GNSS receivers at permanent stations. The consolidated RINEX navigation files are built by cross-checking messages from a large set of individual receivers to ensure that they are valid. This helps prevent the case where a receiver on a site has missing data or generates incorrect values.

The present contribution investigates 43 months of Galileo broadcast navigation data, i.e., from 1 January 2017 to 31 July 2020. We analyze the observed error distribution and characterize the nominal ranging accuracy for each satellite. The probability of satellite failure, $P_{sat}$, and constellation failure, $P_{const}$, is then estimated from the study of the detected satellite failure events. The GPS performance over the same period and over the last 10 years (2010–2020) is also evaluated to compare the Galileo results with a fully deployed and consolidated constellation.

The paper is organized as follows. After the Introduction section, Section 2 provides an overview of the methodology applied in this study, discussing particular details related to the upgrade of the Data Cleansing developed in [12] for GPS to process Galileo navigation data. Section 3 describes the data sets used and identifies some issues related to data processing. Section 4 analyses the observed error distribution, its Gaussian overbounding, and the events over a predefined threshold are identified. The characterization of the observed nominal accuracy is addressed in Section 5, where the mean value and 68th and 95th percentiles are derived. The detected Galileo satellite faults are identified in Section 6 and, based on the observation results, the average $P_{sat}$ and $P_{const}$ are estimated. The paper ends with a summary of the main conclusions.

## 2. Anomaly Monitoring

The methodology applied in this work to identify potential satellite failures is described next, which involves three steps: Data Cleansing, Anomaly Detection, and Anomaly Verification.

### 2.1. Data Cleansing

The broadcast RINEX navigation files collected by the International GNSS Service (IGS) may contain errors or inconsistencies from different sources, such as data login errors due to accidental bad receiver data and/or hardware/software bugs, losses of navigation messages, different transmission time recording, among others.

Data Cleansing is a complex algorithm that builds a cleansed RINEX broadcast navigation file from a wide set of RINEX files of individual receivers distributed worldwide by exploiting the redundancies between them.

The following steps are defined in [12] for GPS broadcast navigation data:

1. Least-Significant Bit (LSB) recovery to remove potential errors in the decoding of navigation messages and convert the values to double-precision floating-point numbers.
2. Classify the GPS URA values to cope with the different URAs appearing in the RINEX files (e.g., some receivers use URA indices instead of URA values, and the same URA index may correspond to three possible values in meters).
3. Duplication removal and majority voting to select the most likely message as the one shared by the largest number of stations, after removing duplicates. In this step, the navigation data are classified as *robust parameters* (most ephemeris and clock parameters) and *fragile parameters* (Transmission Time of Message (TTOM), PRN, URA, Issue of Data Clock (IODC)). The robust parameters are used to identify the candidates of navigation messages. Their associated fragile parameters are then selected as those shared by the largest number of stations.
4. TTOM recovering. The TTOM is not a parameter broadcast in the navigation message. The record given in RINEX navigation files is not the TTOM but the first reception time of the message by the receiver collecting data. The correct TTOM cannot be determined by the oldest one because some IGS receivers may provide an incorrect TTOM older than the actual one. On the other hand, since the IGS stations are not evenly distributed, neither can it be determined simply by the most frequent one. A procedure is then defined to retrieve the TTOM.
5. Minority discard. After the previous steps, few navigation messages can still have errors in their robust parameters, and a uniqueness criterion is applied to select the final candidates.

Upgrading the Cleansing Algorithm to Galileo Broadcast Navigation Data

The Galileo broadcast messages have some particular features that must be taken into account to upgrade the algorithm given in [12] for the GPS to process Galileo data. In short [9]:

- There is only a single Issue of Data Navigation (IOD) to identify the ephemeris, satellite clock correction parameters and SISA, instead of the two IODC and Issue of Data Ephemeris (IODE) of GPS navigation data.
- The following parameters are not linked to the IOD:
  ⚬ The Broadcast Group Delay (BGD) values;
  ⚬ The navigation Data Validity Status (DVS);
  ⚬ The signal Health Status (HS).
  In RINEX navigation files [13], DVS and HS are embedded in the 8 bits of the Satellite Vehicle (SV) health flag for the different signals E1B, E5a, and E5b. Thus, SV health = 0 means that DVS and HS are OK.
  Note: in the GPS, the Total Group Delay (TGD) and SV health are linked to the IODC.
- Galileo broadcast navigation message update [9]:
  ⚬ The typical refresh rate of navigation data ranges from 10 min to 3 h, and each message must be associated with a different IOD;
  ⚬ The maximum nominal broadcast period of a healthy navigation message data set is currently 4 h;
  ⚬ The message Validity Duration (VD) is 4 h.
  Note: the GPS navigation message is, typically, updated every 2 h, with a different IODC, being the validity time given by the Fit Interval (FI), typically 4 h (it can also depend on the IODC value [14]). The validity of the Galileo message is up to 4 h after the Time of Ephemeris (Toe) [9].
- Minority discard step:
  In the case of GPS satellites, candidates are selected according to a uniqueness criterion based on PRN—IODC, i.e., the one confirmed by the larger number of stations having the same PRN—IODC. As the IODC may be occasionally reused by a satellite within the same day, a backup uniqueness criterion based on PRN and Time-of-Clock (Toc) is also applied, i.e., PRN—Toc.
  In the case of Galileo satellites, the previous uniqueness criterion, based on checking the PRN—IODC, cannot be applied. This is because the IOD may be repeated within the same day.
  Thence, with Galileo satellites, for each Data Source’s value in the RINEX file, the uniqueness is based only on the PRN—Toc criterion. This criterion is applied to all robust parameters, except SV health (i.e., DVS, HS) and BGDs, as they are not linked to the IOD.

Finally, all messages containing the selected robust parameters by the previous uniqueness criteria, appearing with any combination of SV health and BGD parameters, are approved and saved in the consolidated broadcast navigation RINEX file.

### 2.2. Anomaly Detection: Space Approach

Anomaly detection follows the “Space Approach” defined in [12], which uses the consolidated broadcast navigation files from the previous Data Cleansing step and precise orbit and clock products provided by IGS, including other sources as well.

The satellite coordinates and clocks are computed from the consolidated broadcast navigation files according to the Galileo OS SDD [9]. The discrepancy between coordinates and clock regarding the precise products is calculated. The orbit error is then projected to the user’s location on the Earth’s surface and combined with the clock error to represent the orbit and clock error at the user level. The Worst-Case User Range Error (WC URE) is calculated, which corresponds to the user location where URE takes the greatest absolute value. The geometric method described in Section 3.5 of [12] (p. 40) has been implemented for the WC URE computation. Anomalies are detected by comparing the WC URE with a threshold defined for a given level of probability (see Section 4.1).

Following [12], adapted to Galileo data, a potential anomaly is declared when all the following conditions are fulfilled:

- The WC URE exceeds the NTE threshold;
- The most recent navigation data set broadcast on a Healthy SIS by the Galileo satellite is used, where Healthy SIS, means:
  The RINEX field SV health [13] is 0, i.e., DVS = “Navigation Data Valid” and HS = “Signal OK”, and SISA ≠ NAPA (NAPA = No Accuracy Prediction Available);
- The Age of Ephemeris (AoE) is smaller than or equal to 4 h Toe, i.e., AoE = t−Toe≤4 h;
- The precise orbits and clocks are available and healthy.
  Note: in the GPS the validity period is given from TTOM and Fit Interval (FI) by Δt = t−TTOM≤FI, where FI is typically 4 h.

A configurable Sampling Rate (SR) of 300 s has been used in this study. Precise orbits at 900 s SR have been interpolated. No interpolation is needed for clocks, as they are available at 300 or 30 s SR. Maximum data holes of 1800 s have been allowed for IGS orbits and 600 s for clocks.

### 2.3. Anomaly Verification: Ground Approach

The anomaly verification follows the “Ground Approach” defined in [12], which uses the RINEX Observation and Navigation files of individual receivers of permanent stations to validate the potential anomalies detected with the Space Approach. The algorithm is based on the following steps:

1. A set of 10 or more active stations having the satellite in view during the whole anomaly event, or as long as possible, is selected. These stations should experience as large anomalous UREs as possible. The algorithm for station selection presented in Section 4.2 of [12] has been implemented in this study.
2. For each selected station (rec), the Instantaneous SIS URE (IURE) is computed from the prefit residual ($prefit_{rec}^{j})$ of the Ionosphere-Free (IF) combination of Galileo C1 and C5 code measurements [15]. That is, for each satellite, *j = 1,…, N* in view from the receiver (*rec*):
  $$prefit_{rec}^{j}\;=\;{\hat{\rho }}_{rec}^{j}-c\cdot {\hat{T}}^{j}+Trop_{rec}^{j}-P_{IF,\;rec}^{j}$$
  where ${\hat{\rho }}_{rec}^{j}$ and $\hat{T}$ are the geometric range and the satellite clock offset computed with the broadcast ephemeris, $Trop_{rec}^{j}$ is the tropospheric delay estimated using the UNB-3 nominal model and the simple mapping function implemented in the gLAB tool [16], and $P_{IF}{}_{rec}^{j}$ is the IF combination of unsmoothed code measurements. Satellites below 5 deg of elevation are excluded.
  The IURE for the anomalous satellite (*sat*) is then computed as:
  $$IURE\;=\;prefit_{rec}^{sat}+c\cdot {\hat{T}}_{rec}$$
  where ${\hat{T}}_{rec}$ is the receiver clock offset estimated as the weighted average of the prefit residuals of all satellites in view, excluding the anomalous satellite (*sat*) (see Equation (4.7) in [12]).
  A configurable sampling rate of 300 s has been used in this study.
3. Following [12], the Galileo satellite is set as “anomalous” when all the following conditions are fulfilled:
  - The IURE exceeds the NTE threshold;
  - The most recent navigation data broadcast on a Healthy SIS by the Galileo satellite are used, where Healthy SIS means:
    The RINEX field SV health is 0, i.e., DVS = “Navigation Data Valid” and HS = “Signal OK” and SISA ≠ NAPA.
  - The broadcast navigation message is within its validity time, i.e., AoE = t−Toe≤4 h;
  - The signal was tracked with an acceptable SNR, i.e., the RINEX SNR flag value ≥4.

**Figure 1:**
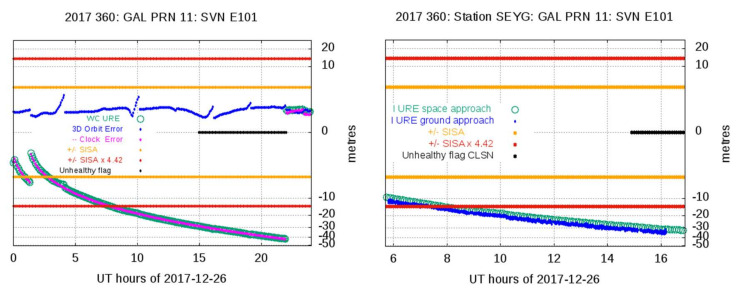
Example of anomaly detection and verification for the Galileo satellite E101 on 26 December 2017, using the Not-to-Exceed (NTE) = 4.42 × SISA threshold. The left plot illustrates the Anomaly Detection from the Space Approach, and the 3D orbit and clock errors are shown in blue and pink, respectively. The green circles correspond to the Worst-Case User Range Error (WC URE). The Signal-in-Space Accuracy (SISA) value is in yellow, and the NTE threshold in red. The unhealthy flag from the cleansed Receiver Independent Exchange (RINEX) navigation file is in black. The right plot illustrates the Anomaly Verification from the Ground Approach, using measurements from the station SEYG. The Instantaneous SIS URE (IURE) values computed from the station SEYG measurements are shown in blue, and the IUREs from the Space Approach are in green. The unhealthy flag from the cleansed RINEX navigation file is shown in black. The SISA values and the NTE threshold are shown in yellow and in red, respectively. The *y*-axis is in a cubic root scale.

Figure 1 shows an example of Anomaly Detection, with the Space Approach in the left plot and Anomaly Verification with the Ground Approach in the right plot. This example corresponds to the event experienced by the Galileo satellite E101 on 26 December 2017. An NTE threshold of 4.42 × SISA is used. As depicted in the left plot, due to a large clock error (in pink), the WC URE (green circles) goes over the NTE at 07:40 of 26 December 2017, with the satellite set as healthy. This anomalous condition ends when a new navigation message with an unhealthy condition (black line) is received at 15:00. The orbit error (in blue) is kept under the NTE threshold. This potential event is confirmed by the Ground Approach shown in the right plot, using measurements from the station SEYG (in Seychelles islands). The IURE values computed from Space and Ground approaches are shown in green and blue, respectively. The unhealthy condition from the RINEX flag shown in black corresponds to the consolidated (cleansed) RINEX file.

### 2.4. Decision Criterion

The potential SIS anomaly is considered “true” when none of the selected receivers show a nominal IURE, at least one receiver from the selected set shows an anomalous IURE, and the rest are unable to track the satellite during the anomalous event. On the contrary, an anomaly is considered “false” when none of the selected IGS receivers shows anomalous IURE, at least one of the receivers from the selected set shows nominal IURE, and the rest do not track the satellite. The case where, at the same time, there are receivers that present anomalous and nominal IURE is considered “paradoxical” and requires manual intervention. The satellite is considered “untracked” when the selected receivers with the anomalous satellite in view cannot track it. In this case, the anomaly is very likely to be false [12].

## 3. Data Sets

The previous data cleansing, anomaly detection, and verification procedures have been applied over 43 months of F/NAV Galileo navigation data, from 1 January 2017 to 31 July 2020.

Worldwide RINEX-2/3 Navigation files have been gathered from several public domain servers, such as CDDISA, EUREF and ESNG, avoiding repetitions of stations. The already-compiled RINEX navigation files named “brdc, brdm or auto” are not used to guarantee “one station one vote.” Dual-frequency RINEX-2/3 Observation files at a 1 Hz sampling rate have also been gathered from IGS servers.

Precise orbit and clock products from the Multi-GNSS Experiment (MGEX) (CODE products) [17] have been used to check the broadcast navigation data (with orbits at 900 s until 5 August 2017 and at 300 s later, and clocks at 300 s until 11 August 2017 and at 30 s later).

The Antenna Phase Centers (APCs) and the System Time used in the IGS products is different from those used in the broadcast navigation data. Thence, the Antenna Exchange Format (ANTEX) file provided by the European GNSS Service Center [18] has been used, which has the same APCs as in the broadcast Galileo ephemeris. Some update has been necessary for the ANTEX reading, as these files use a different reference than IGS ANTEX files.

To align the IGS clocks to the Galileo system time, the IGS clocks have been corrected first for the difference (∆APC) between Galileo broadcast and IGS APCs. The epoch-wise trimmed mean of the difference between the IGS (∆APC corrected) and broadcast clocks has been computed to estimate the difference between Galileo and IGS reference times (∆T). This trimmed mean is calculated after removing the 20% of data above and below the epoch-wise median. Finally, the IGS (∆APC corrected) clocks are aligned with the Galileo system time by correcting with ∆T.

**Figure 2:**
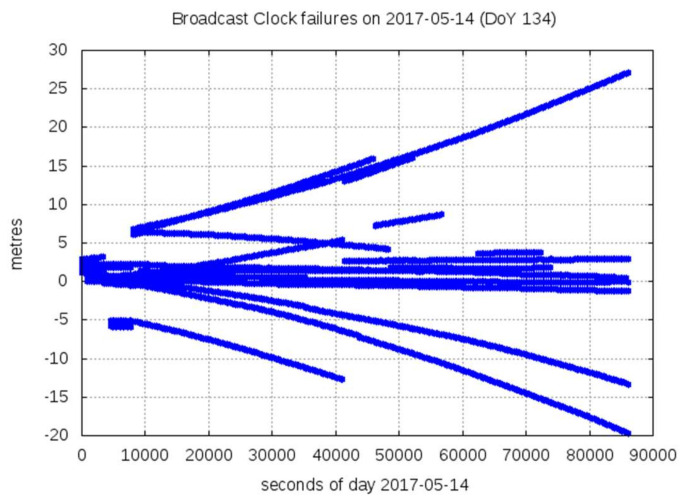
Simultaneous events occurring in Galileo broadcast clocks on 14 May 2017. Several satellites experience large broadcast clock errors with respect to the precise clock determinations because the navigation messages were not refreshed.

Although this trimmed mean can protect against clock outliers due to one or few faulty satellites, the estimation of ∆T with the previous approach can be affected by simultaneous satellite events, as experienced on 14 May 2017 (see Figure 2).

For the GPS satellites, the National Geospatial-Intelligence Agency (NGA) precise products [19] have been used, which requires neither any APC correction nor time alignment. The sampling rate of these products is 900 s until 27 February 2012 and 300 s afterwards.

## 4. Observed Error Distribution

This section analyses the observed error distribution in the coordinates and clocks of Galileo and GPS satellites, computed from the F/NAV and LNAV broadcast navigation messages, respectively.

**Figure 3:**
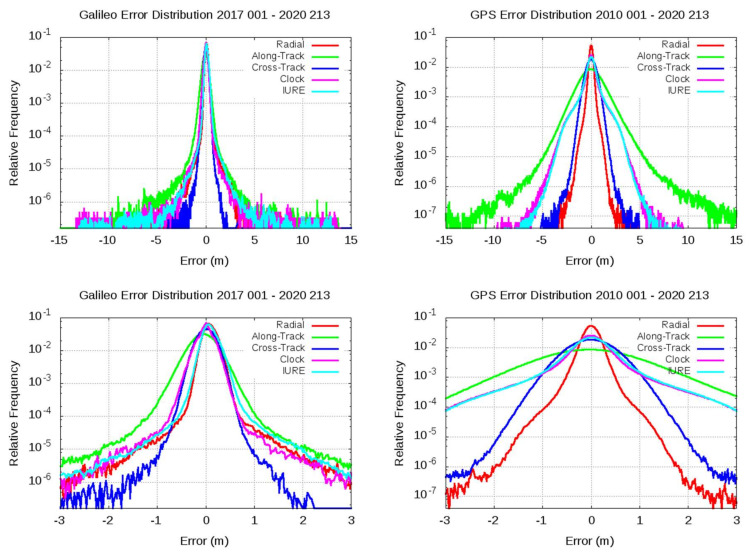
Relative frequency histogram for the observed error distribution of broadcast navigation data from aggregated data of all satellites (bin size: 2 cm). The plots in the left column are for Galileo F/NAV from 1 January 2017 to 31 July 2020. The plots in the right column are for GPS LNAV data from 1 January 2010 to 31 July 2020. The plots show the radial (red), along-track (green), cross-track (blue), clock (pink), and IURE (cyan) errors. Bottom plots are an *x*-range zoom of top plots. The eccentric satellites E201 and E202 are excluded.

The plots of Figure 3 show the relative frequency histogram for the aggregate total radial (red), along-track (green), cross-track (blue), clock (pink), and IURE (cyan) errors. The plots in the left column involve more than 3.5 years of Galileo F/NAV navigation data, i.e., from 1 January 2017 to 31 July 2020. The plots in the right column are for the GPS satellites and LNAV message and contain more than 10 years’ data, i.e., from 1 January 2010 to 31 July 2020. Although larger periods are also available for GPS, it is unclear if they would represent the current state of the system [11]. The bottom plots are a zoom of top plots to better see the distribution peaks.

A sharper distribution is found for Galileo satellites compared with the GPS histogram. The radial and cross-track errors are the error components more tightly distributed in both Galileo and GPS. Similar distributions are found for Galileo radial and clock errors, leading to a similar pattern for IURE. Moreover, a small bias appears in the radial error and IURE (see left column, bottom plot). This bias is quantified in Section 5, when analyzing the observed nominal accuracy. No bias is observed in the clock error, although it may have been absorbed, in some way, by the clock alignment process. In the GPS, the IURE is clearly dominated by clock error, with fairly overlapping patterns. Cross-track error is sharper in the Galileo than in the GPS, whereas along-track exhibits a larger spread in both Galileo and GPS data. No remarkable biases are found in the GPS error distributions.

### 4.1. Identification of Potential SISE Events

The SISE values, measured as the instantaneous maximum projected ranging errors at the worst user location, i.e., WC URE, are analyzed this section and the next. We start identifying first potential events having anomalous SISE values and then we will analyze the SISE overbounding by a Gaussian distribution.

As stated in the OS SIS ICD [20], the “SISA is a prediction of the minimum standard deviation (1*σ*) of the unbiased Gaussian distribution, which overbounds the SISE predictable distribution for all possible user locations within the satellite coverage area.”

**Figure 4:**
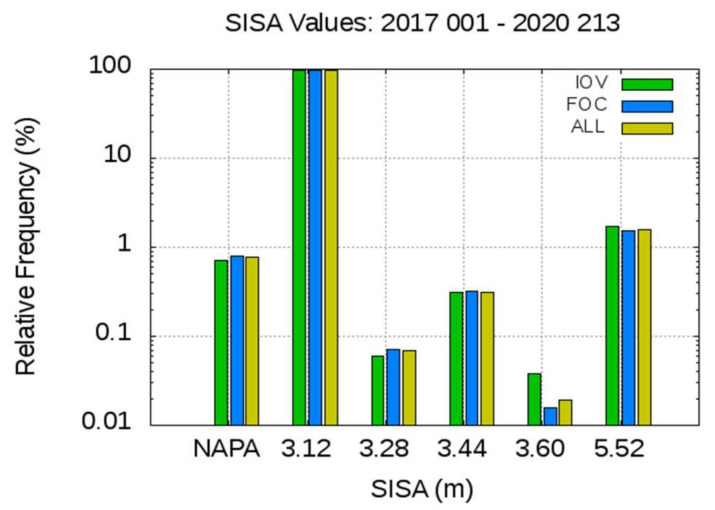
Relative frequencies of the Broadcast SISA values for In-Orbit Validation (IOV), Full Operational Capability (FOC), and Aggregate All satellites (ALL). More than one order of magnitude of difference is found between the most frequent SISA value, 3.12 m, and the other broadcast values. No Accuracy Prediction Available (NAPA) is broadcast in less than 1% of cases. The eccentric satellites E201 and E202 are excluded.

Figure 4 depicts the relative frequency of the different broadcast SISA values for the IOV and FOC satellites and across the whole constellation, excluding the eccentric satellites E201 and E202. As depicted, the most frequent SISA value (more than 97%) is 3.12 m in these three satellite sets, and NAPA is broadcast in less than 0.8% of cases.

The methodology of Anomaly Monitoring presented in Section 2 is used next to identify anomalous behaviors in Galileo and GPS satellites. The same threshold as GPS with IAURA is used for Galileo with SISA to identify potential events, i.e., NTE = 4.42 × SISA.

**Figure 5:**
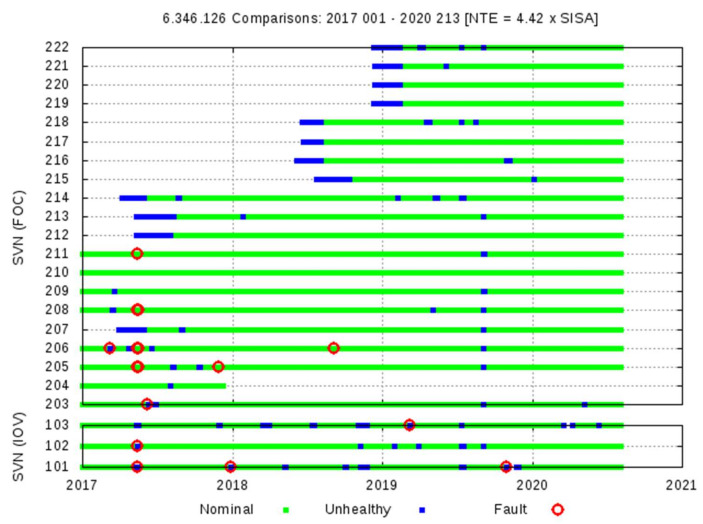
Galileo F/NAV, from 1 January 2017 to 31 July 2020. Summary of observations for each Galileo satellite. Green indicates valid observations, blue indicates the satellite was unhealthy, and red circles indicate events exceeding the 4.42 × SISA threshold. Consolidated broadcast RINEX files from the Group of Astronomy and Geomatics (gAGE) and MGEX precise products have been used. The Galileo eccentric satellites E201 and E202 are excluded.

A summary of the identified Galileo F/NAV events is given in Table 1 and Figure 5. Regarding Table 1, the detections with the Space Approach are shown on the left and the verification results with the Ground Approach on the right. The Satellite Vehicle Numbers (SVNs) E1XX corresponds to the IOV satellites and E2XX to the FOC satellites (see Figure 5).

Ten different Galileo satellite events exceeding the NTE = 4.42 × SISA threshold are found in 2017 (involving the satellites E101, E102, E203, E205, E206, E208, and E211), only one satellite event is identified in 2018 (E206), and two more satellite events in 2019 (E101 and E103). These detections have been confirmed by the Ground Approach algorithm (Algorithm Decision column) or set as Paradox. The last column of this table shows the Final Decision based on further analysis. Multiple satellite detections appear on 14–15 May 2017.

**Figure 6:**
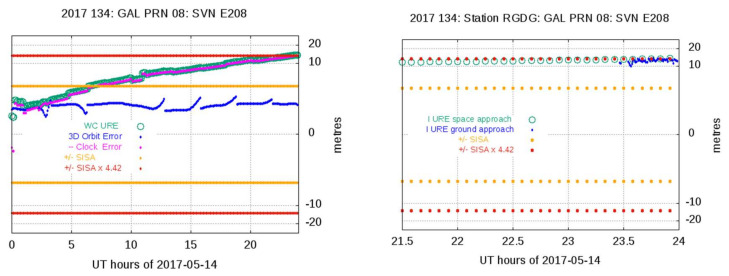
Space (left plot) and ground (right plot) analysis of the Satellite Vehicle Number (SVN) E208 event on 14 May 2017. The left plot shows the space approach with a WC URE just reaching the detection threshold of 4.42 × SISA. The right plot shows the Ground Approach results for the only station of Table 1, RGDG, detecting the anomaly. As depicted, the WC URE values are reaching the threshold at the end of the day. The *y*-axis is in a cubic root scale.

It is worth mentioning the detection was found with the space approach for SVN E208 at the end of 14 May 2017 (see Table 1). The Ground Approach algorithm declares this event as “Paradoxical” because only one station, the RGDC, Rio Grande (Argentina), in the selected set of 50 stations, exhibits abnormal behavior during the analyzed time interval, while seven stations are in nominal mode. The other analyzed 42 stations untracked the signal. Figure 6 shows, at the left, the plot of space approach with the WC URE over the threshold at the end of the day. The right plot shows the Ground Approach plot for the station RGDG, where the ground IURE, blue dots, reaches the 4.42 × SISA threshold, indicated by red dots. Although the other seven stations tracking the satellite are in a nominal condition on this day, many of them detect the anomalous condition a few minutes after on the next day. In fact, this is the same event involving both 14 and 15 May 2017.

The multisatellite events detected on 14–15 May were produced by hardware equipment failure in the ground segment of Galileo. As a result, the navigation message for all satellites was not refreshed. The cause root of this failure was identified, the equipment was replaced, and the services were recovered to their nominal levels at 12:44 of 16 May 2017 (see NAGU2017015 [21]). Figure 2 depicts the large error experienced by several of these satellites during this event on 14 May 2017.

As the consolidated (cleansed) RINEX navigation files are a critical input for the Space Approach anomaly detection, and in order to improve the reliability, a double-check has been performed using the consolidated Galileo RINEX navigation files provided by Centre National D’Etudes Spatiales (CNES), cleansed with the “Galileo Ephemeris Consolidation and Control Analysis” (GECCO) software (CNES, Toulouse, France) [22]. The GECCO cleansed RINEX files are available at the CNES server [23].

**Figure 7:**
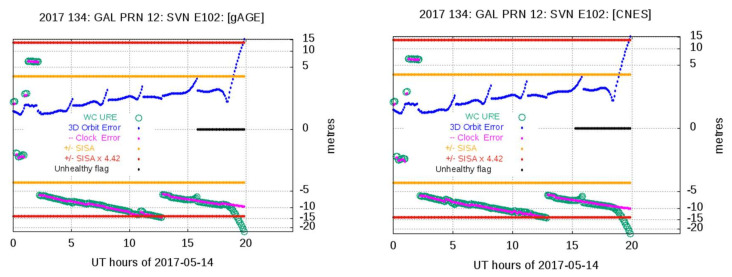
Example of anomaly detection using the consolidated RINEX navigation files from gAGE (left plot) and from Galileo Ephemeris Consolidation and Control Analysis (GECCO) (Centre National D’Etudes Spatiales (CNES)) (right plot). The *y*-axis is in a cubic root scale.

All true events detected with the gAGE cleansed RINEX navigation files were also detected with the GECCO cleansed files. Figure 7 shows an example of anomaly detection with the Space Approach using RINEX cleansed files from gAGE (left plot) and from GECCO (CNES) (right plot), with similar results.

The 29 October 2019 event detected in the Galileo IOV E101 satellite (see Table 1) is described next in detail, as it will be relevant in Section 6 when estimating the observed satellite fault probability.

#### Galileo IOV E101 Satellite Event on 29 October 2019

**Figure 8:**
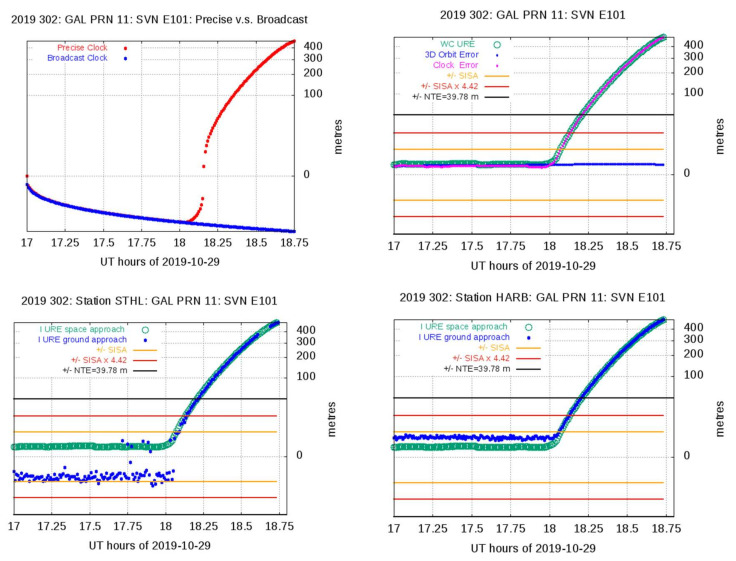
View of the IOV E101 satellite fault on 29 October 2019. The left plot in the first row depicts, in red, the precise clock values estimated by the gAGE/Universitat Politècnica de Catalunya (UPC) processing facility and, in blue, the broadcast clock (values shifted to zero at 17:00 h). The right plot in the first row shows the anomaly detection by the Space Approach. A satellite fault is declared when WC URE reaches the NTE = 39.78 m threshold. The plots in the second row show the anomaly verification by the Ground Approach, using the station STHL (left plot) and HARB (right plot). Horizontal lines indicate +/− SISA, yellow; +/−4.42 × SISA, red; and NTE, black. The *y*-axis is in a cubic root scale.

A major service failure was experienced by the IOV satellite E101 on 29 October 2019. A brief description of this event and its detection is depicted in Figure 8.

At 17:31:30 29 October 2019, a F/NAV message with IOD = 8 is received, indicating that the satellite was Healthy SIS, i.e., DVS = “Nav. Data Valid” and HS = “Signal OK” and SISA = 3.12 m. The next message is not received until 18:43:30 on the same day.

At approximately 18:00, the satellite clock begins to experience a large drift. This is depicted by the precise clock determination shown by the left plot in the first row of Figure 8. This behavior cannot be reproduced by the broadcast clock that follows a linear drift (see the blue line in the same plot). The precise clocks estimated with the gAGE/UPC Processing Facility [24] have been used in this plot, but the same drift is found in the precise clocks from CODE, GFZ, or CNES.

About 12 min later, the WC URE exceeds the threshold NTE = 39.78 m considered in the Milestone 3 report [7] to declare a major service failure (see, for instance, the right plot in the first row of Figure 8). The anomalous condition ends at 18:43:30 after the reception of a new message with IOD = 15, with DVS = ”Navigation Data Valid” and HS = ”Signal OK,” but SISA = NAPA, which means that OS SIS status was set to MARGINAL. The satellite was not declared as Healthy SIS up to several days after.

This anomaly detection by the Space Approach is illustrated in the right plot at the first row of Figure 8. The clock error drift dominates the WC URE, reaching the NTE threshold at about 18:12. The orbit error is well maintained at its nominal value. The second row of Figure 8 illustrates the verification of this anomaly by the Ground Approach, using the station STHL, Santa Helena island (UK) (left plot) and station HARB, Hartebeesthoek (South Africa), (right plot). A total of 50 stations have been used to verify this anomaly (see Table 1), with the anomaly being confirmed by 18 of them. The other 32 were not tracking the satellite at that time.

### 4.2. GPS Satellites: Events Exceeding the 4.42 × IAURA Threshold

**Figure 9:**
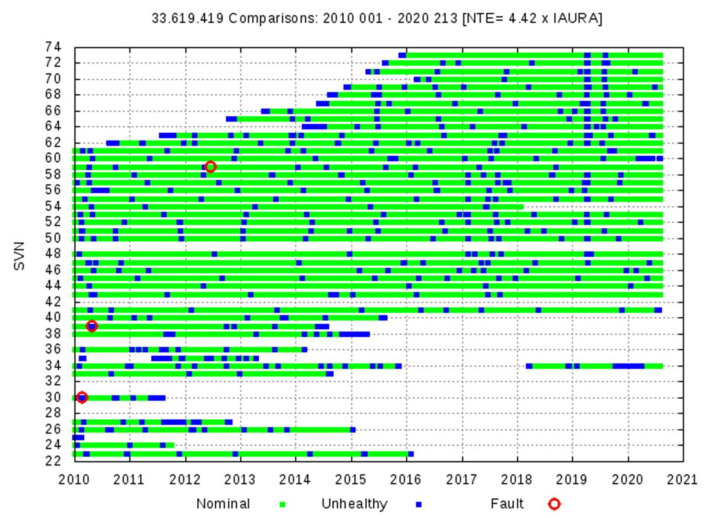
GPS LNAV, from 1 January 2010 to 31 July 2020. Summary of observations for each GPS satellite. Green indicates good observations, blue indicates the satellite was unhealthy, and red circles indicate events exceeding the NTE = 4.42 × IAURA threshold. Consolidated broadcast RINEX files from gAGE, and NGA precise orbits and clocks are used.

Figure 9 and Table 2 summarize the analysis performed on GPS satellites for the period dating from 1 January 2010 to 31 July 2020, strictly applying the methodology given in [12]. In this period of more than 10 years, only three events are recorded where the WC URE exceeds the threshold NTE = 4.42 × IAURA, totaling 50 min of failure. The first two failures occurred on 22 February and 25 April 2010, and the third occurred on 17 June 2012. No more failures until 31 July 2020 have been detected. The analysis and validation of these failures are detailed in [11], and we will not address them any further.

### 4.3. Signal-in-Space Error Overbounding

As indicated in the introduction, the nominal satellite ranging accuracy is typically characterized by a Gaussian distribution that overbounds the true distribution out to some probability level [25,26]. The model also assumes that much larger errors can be experienced than would be expected according to the Gaussian distribution, but with a very low probability. This small probability corresponds to the fault likelihood [11].

**Figure 10:**
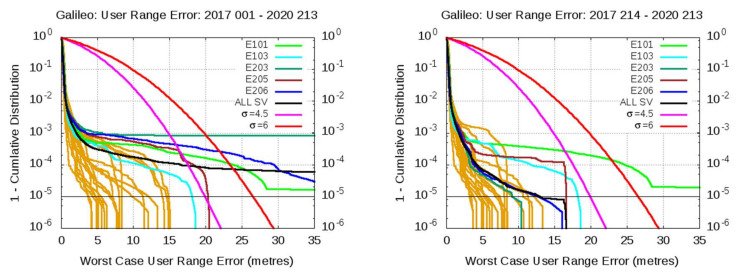
One minus the empirical Cumulative Distribution Function (1-CDF) of the maximum projected ranging errors of Galileo F/NAV broadcast navigation data. The left plot shows the aggregated values from 1 January 2017 to 31 July 2020. The right plot excludes the first six months of data, comprising the period from 1 August 2017 to 31 July 2020. Satellites not bounded by the Gaussian distributions, with *σ* = 4.5 and *σ* = 6 m curves, are shown in pink and red, respectively.

An overbound of SISE by a zero-mean Gaussian distribution with σ = 4.5 m for the aggregated distribution from all satellites is found in [27], after extrapolation to what is expected when the Galileo constellation reaches the FOC. A slightly higher URA value of 6 m is proposed by ICAO NSP [10] as a conservative overbound of the actual SISE to have some additional margin for Horizontal ARAIM (H-ARAIM) Galileo dual-frequency users. These two sigma values are assessed in Figure 10 for the observed SISE overbounding over two time intervals, the full period of 43 months and the last three-year window, i.e., excluding the first six-month period. This analysis is based solely on the experimental error distribution, without any extrapolation to the FOC.

The plots of Figure 10 show the One-minus empirical Cumulative Distribution Function (1-CDF) of the WC URE. The pink and red lines indicate the expected values for a Gaussian distribution with a zero mean and standard deviations σ = 4.5 and σ = 6 m, respectively. The left plot comprises the full period, from 1 January 2017 to 31 July 2020. Satellites not bounded by any of the two Gaussian distributions, with σ equals to 4.5 or 6 m, are indicated by different colors, i.e., E101, E103, E203, E205, and E206. The aggregated 1-CDF for all satellites is shown in black. Several of these satellites experienced anomalous events during the firsts six months of operation after the IS OS was declared, affecting the CDF behavior. The root cause of these events has been analyzed in detail by the CSP, and most of them are not considered representative of the FOC of Galileo [27]. In fact, according to the Galileo Project Office, only the events of E203, on 6 June 2017, and E101, on 29 October 2019, are considered representative of the FOC [5] (see Section 6.3).

The right plot of Figure 10 shows the same 1-CDF, excluding the first six months of data, where many of the abovementioned events occurred, i.e., from 1 August 2017 to 31 July 2020. This time-window eliminates most, but not all, of the abovementioned events that occurred. As depicted, the two Gaussian distributions with σ = 4.5 and σ = 6 m overbound all satellites, except E101. Moreover, the aggregated 1-CDF, incorporating all satellites, in black, is well bounded below the probability level 1 × 10^−5^.

It is worth to say that, although the system is assumed to be stationary, this hypothesis is not entirely true. In fact, the ground segment software is updated and improved over time, and the satellite designs are refined with enhanced capabilities. Therefore, the system is expected to evolve toward a better performance along time, and stationarity can be assumed as a conservative hypothesis. It should be also noted that, whether or not a satellite exceeds the Gaussian distribution, at a given probability level, depends on the magnitude of the fault and on the total amount of data available to the satellite, regarding to the fault duration. For instance, in the case of the E206 event of the Galileo satellite (5 September 2018, see Table 1), the amount exceeding the threshold and duration of the event was not long enough, compared with the total amount of data, to strongly affect the 1-CDF overbounding (see Figure 10, right plot, Table 1 and Figure 5).

**Figure 11:**
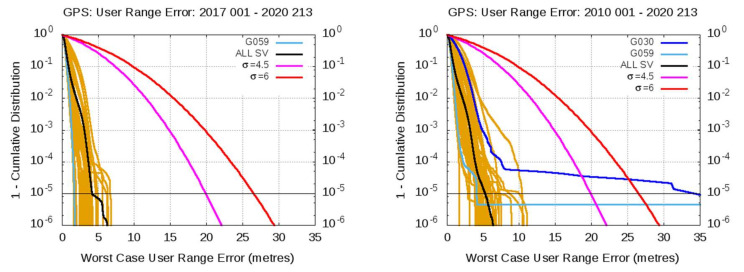
The same plots as in Figure 10, but for GPS LNAV broadcast navigation data in the periods 1 January 2017 to 31 July 2020, in the left plot, and 1 January 2010 to 31 July 2020, in the right plot.

Figure 11 shows the same plots as Figure 10 but for the GPS satellites over the same 43 months of data period, from 1 January 2017 to 31 July 2020, at the left plot, and about over a 10-year period, from 1 January 2010 to 31 July 2020, in the right plot. In the first case, all satellites largely fall under the Gaussian distributions depicted by the pink and red lines. In the right plot, only the GPS satellite G030 goes beyond the red line above the 1 × 10^−5^ probability level. Satellite G059 also crosses the red line, but well below 1 × 10^−5^. As shown in Table 2 and Figure 9, although this satellite experienced a fault event (17 June 2012) of about 20 min of duration, and with a WC URE reaching up to 451.5 m, it falls below the 1 × 10^−5^ level, due to the large amount of valid data available of this satellite. This is not the case of the event that occurred on satellite G030 (22 February 2010), which, with a similar duration and with a WC URE of only 42.9 m, impacts more to the CDF, exceeding the Gaussian distributions above the $10^{-5}$ level, due to the short amount of valid data available in the analyzed period.

Having in mind the previous considerations, it is important to point out that the Galileo results are based on a reduced amount of data, about three years and a half, and some of the events experienced (identified in the previous Section 4.1) do not reflect the FOC configuration of the system. The results must then be consolidated with large observational data.

## 5. Observed Nominal Accuracy

The observed nominal accuracy of Galileo data is derived by excluding the tails of the SISE distribution analyzed in Section 4.3. Thence, Nominal Condition in Galileo is assumed when the following conditions are met:

- The WC URE is under the 4.42 × SISA threshold;
- The most recent navigation data set broadcast on a Healthy SIS by the Galileo satellite is used, where Healthy SIS means:
  The RINEX field SV health is 0, i.e., DVS = “Navigation Data Valid” and HS = “Signal OK” and SISA ≠ NAPA;
- Broadcast navigation message is within its validity time, i.e., AoE = t−Toe≤4 h;
- The precise orbits and clocks are available and healthy.

Table 3 shows, for the Galileo F/NAV navigation data, the overall mean value, 68th and 95th percentiles, and the sigma value for the Galileo radial, along-track, cross-track, clock, WC URE, and IURE. The values are given for each individual satellite, grouped by block and aggregate total. In the case of IURE, the values have been estimated over 20 points spread evenly on the Earth, derived from the vertices of a regular dodecahedron [12]. The analyzed period comprises from 1 January 2017 to 31 July 2020. The GPS nominal accuracy from LNAV navigation message over the same period is given in Table 4 for comparison.

**Figure 12:**
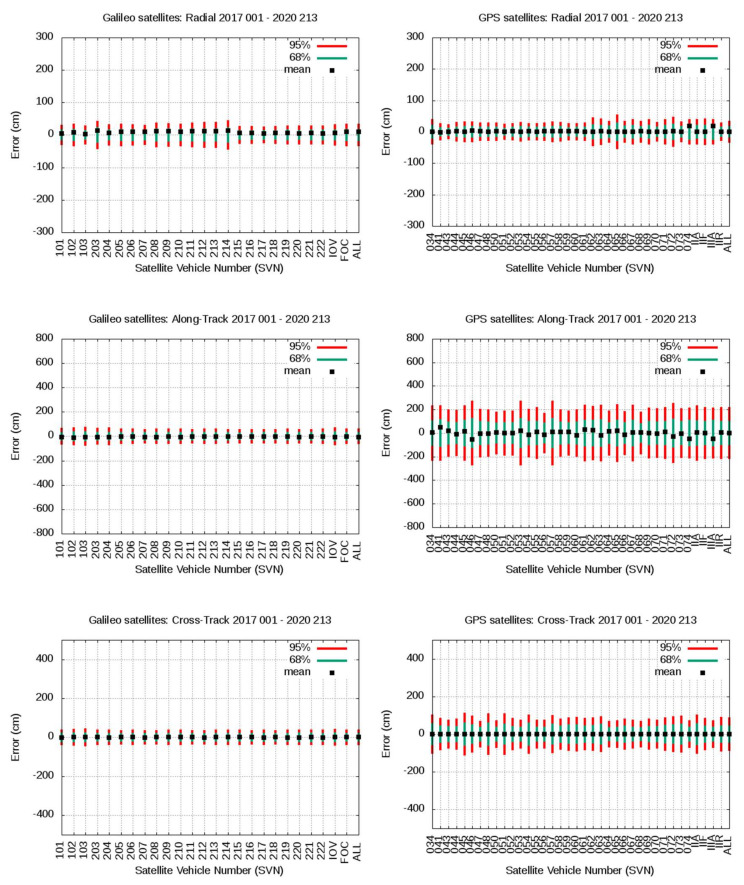
From top to bottom the plots are for Radial, Along-track and Cross-track. Left column plots are for Galileo F/NAV and right column plots for GPS LNAV navigation data. The same vertical range is used for Galileo and GPS satellites. The SVNs are in the horizontal axis. Each plot shows the mean value and 68th and 95th percentiles associated to Table 3 and Table 4.

**Figure 13:**
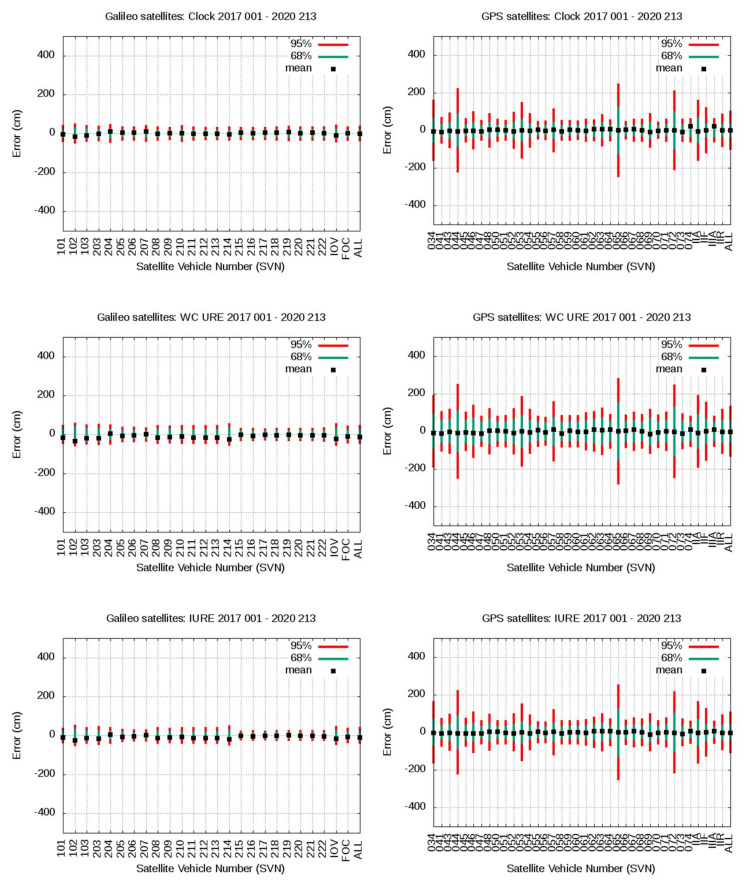
From top to bottom the plots are for Clock, WC URE and IURE. Left column plots are for Galileo F/NAV and right column plots for GPS LNAV navigation data. The same vertical range is used for Galileo and GPS satellites. The SVNs are in the horizontal axis. Each plot shows the mean value and 68th and 95th percentiles associated to Table 3 and Table 4.

Figure 12 and Figure 13 show a compact view of the mean value and 68th and 95th percentiles associated with Table 3 and Table 4. The plots in the left column are for Galileo satellites, and plots in the right column for GPS satellites. From top to bottom, plots in Figure 12 are for radial, along-track, cross-track values, and plots in Figure 13 are for clock, WC URE and IURE values. The consolidated gAGE RINEX navigation files for Galileo and GPS have been used in this assessment.

As it can be seen in Figure 12, Figure 13 and Table 3, except for the radial component, the Galileo satellites show much smaller percentile values than the GPS satellites. Nevertheless, the mean bias appearing in the radial component of IOV and FOC Galileo satellites, of 5.6 and 9.7 cm, respectively, is significant. The largest biases are found in the FOC satellites, mainly on E203 to E214, reaching up to more than 10 cm. It is worth mentioning that the ANTEX files igs14_2118.atx from IGS and GSAT_2023.atx (with its associated Antenna Reference Points) from the European GNSS Service Center [18] have been used, and only discrepancies in their APCs were identified on satellites E215 to E222, which are those experiencing the smaller biases in the FOC satellites. No discrepancies are found in the APCs for the IOV satellites. Thence, as the larger biases are associated with satellites having the same APCs in both ANTEX files, the abovementioned biases are not due to any mismatch between the APCs used in the IGS products and Galileo broadcast orbits for these satellites. In spite of these biases, the 68th and 95th percentiles and the standard deviation in the radial component error are of the same order as those of GPS (see Table 3 and Table 4). This positive bias in the radial component could be partially linked to the accuracy of the MGEX (CODE) reference products for Galileo satellites at the level of 5 cm [28], but it deserves further studies.

The clock alignment applied for Galileo satellites can absorb a global bias in the clock error, and this is probably the reason for having only −0.3 cm of total mean clock error in the last row of Table 3. Satellites E102, E103, E204, and E207 experience clock biases of about 10 cm or more. In spite of these biases, again, the 68th and 95th percentiles and sigma are similar, or even smaller, than in GPS. The combined bias in the radial component and clocks is translated to the WC URE, exhibiting global values of 22.7 and 10.7 cm for IOV and FOC Galileo satellites, but the 68th and 95th percentiles and sigma are smaller than those of GPS.

The along-track error component of Galileo satellites is several times smaller than the GPS, as depicted by the four statistics shown in Table 3 compared with Table 4. The cross-track component also shows smaller error figures than those of the GPS.

Finally, and as expected from the previous results, the Galileo IURE values, in the right of Table 3, shows a mean bias highly correlated with the WC URE values, while the 68th and 95th percentiles and sigma are, again, smaller than in the GPS.

Last but not least, in order to have a more robust estimation of GPS nominal accuracy, Table 5 shows the values computed over the more than the ten-year period considered before, from 1 January 2010 to 31 July 2020. As shown, values quite similar to those in Table 4 are obtained.

## 6. Observed Fault Probabilities

The observed probabilities of satellite ($P_{sat}$*)* and constellation ($P_{const}$) failures are estimated next following the definitions of [4]. The expected satellite Fault Rate (R), given by k events over the interval T, can be estimated by the expression:

(1) $$E(R|k)\;=\;\frac{k+1/2}{T}$$

where T is the aggregated total signal of valid hours, i.e., with signals indicating that they were healthy (aggregated for all satellites). The probability of a satellite fault ($P_{sat}$) is the fault rate multiplied by the Mean Time to Notify (MTTN) the user, i.e., the delay between the event onset and the average time for the system to notify such event to the user:

(2) $$P_{sat}\;=\;E(R|k)\times \mathrm{MTTN}$$

Derivation of these formulae can be found in [4], where it is assumed that the probability of faults follows a Poisson distribution and the a priori probability of R is approximated by a distribution $f\left(R\right)\propto 1/\sqrt{R}$ between 0 and $R_{max}$.

A methodology to estimate the MTTN in Galileo is summarized in [27], where 60 min for ATTM are expected for the future configuration of the Galileo system in the FOC. Further results from ICAO NSP [10], considering improved monitoring capabilities on the ground and tuning and mentioning barriers, expect to reduce this value.

The satellite fault events and their duration have been identified applying, again, the methodology of Section 2, but considering the two aforementioned thresholds:

(1) NTE = 4.42 × 9 = 39.78 m threshold, according to the already indicated Galileo commitments [7,9];
(2) NTE = 4.17 × 6 = 25.04 m threshold recently proposed to the ICAO NSP on April 2020 [10].

In the last subsection, the results are extrapolated to the FOC of the Galileo program.

### 6.1. Observed Fault Probabilities Based on NTE = 39.78 m

According to the Milestone 3 report [7], the target H-ARAIM service level can be established based on GPS and Galileo with the following contribution from Galileo:

- URA (overbound of SISE) lower than 9 m;
- $P_{sat}$ lower than 1 × 10^−5^/sat;
- $P_{const}$ lower than 1 × 10^−4^.

The NTE = 4.42 × *URA* = 39.78 m will then be used for Fault Detection.

**Figure 14:**
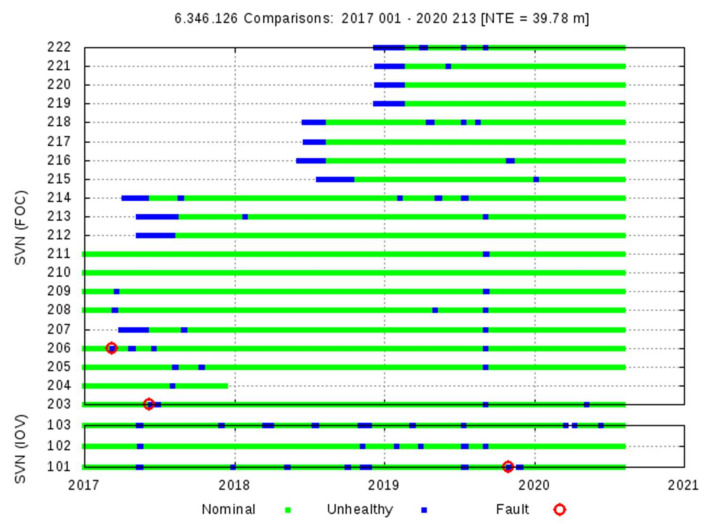
The same plot as in Figure 5, but considering the NTE = 39.78 m threshold.

An overview of detection results using NTE = 39.78 m, is given in Table 6 and depicted in Figure 14. Two satellite failures are found in 2017, the E206 (on 7 March) and E203 (on 6–7 June), and only one satellite failure in 2019, the E101 (on 29 September) (see details of this last event in Section 4.1).

Table 7 summarizes the number of satellite faults, the cumulative duration of detected faults, and total of signal valid hours in each year from 2017 to 2020. The first row, after the header, specifies the values from 1 August to 31 December 2017. The last row indicates the values from 1 January to 31 July 2020. Results are shown for the IOV and FOC satellites and across the whole constellation. These values are from the duration column in Table 6.

Table 8 shows the cumulative results from Table 7 for the two previously considered periods, from 1 January 2017 to 31 July 2020 (first row after the header) and for the last three-year time window, from 1 August 2017 to 31 July 2020 (in the last row). It is worth mentioning that in this last period, i.e., excluding the firsts six months of data, only one satellite fault is found, which was experienced by the IOV satellite E101 on 29 October 2019. 

A simple experimental estimation of MTTN can be made from the observed averaged duration of faults, but it is worth mentioning that this can only be seen as a rough estimate of this value. To be conservative, in the numerical application, we then use as MTTN the highest value between this averaged duration of faults and the 60 min given in [27], see Table 8.

As shown, the obtained results for the observed fault probability over the last three-year time window are very promising, as the estimated value of $P_{sat}$ = 3.2 × 10^−6^/sat given in Table 8 is well below the previous commitment of 1 × 10^−5^. This value increases to 5.8 × 10^−5^/sat when considering the full period of 43 months, but as already commented, most of the faults experienced during the first half of 2017, and even others detected after this period, are considered not representative of what is expected in the FOC configuration [27].

No Wide Faults, i.e., affecting more than one satellite simultaneously, appear when considering the NTE = 39.78 m threshold. Nevertheless, taking into account the reduced size of data, a conservative value of $P_{const}$ = 1 × 10^−4^ can be used [11].

### 6.2. Observed Fault Probabilities Based on NTE = 25.04 m

The Galileo program established a dedicated process involving the main actors (EC, ESA, and GSA), which analyze the Galileo performance to support the definition of the ARAIM concept and relative standards. The conclusions presented in the ICAO NSP on April 2020 consider the following values for Galileo [10]:

- URA (overbound of SISE) lower than 6 m;
- $P_{sat}$ lower than 3 × 10^−5^/sat;
- $P_{const}$ lower than 1 × 10^−4^.

The NTE = 4.17 × *URA* = 25.04 m will then be used for Fault Detection.

As shown in Table 1, there is an additional event out of those of Table 6 having WC URE over the NTE = 25.04 m. The IOV satellite E101 experienced this event on 26 December 2017. The WC URE was over this NTE for about 20 min, reaching a maximum value of 27.2 m.

The estimated mean conditional fault rate $E\left(R|k\right)$ and $P_{sat}$ for this more stringent threshold can be found in Table 9. As shown, $P_{sat}$ = 5.3 × 10^−6^/sat is estimated when considering the last three years window, and MTTN = 1 h, which is still about one order of magnitude below the 3 × 10^−5^/sat value. Moreover, again, no Wide Faults appear when considering this NTE = 25.04 m threshold, and the conservative value of $P_{const}$ = 1 × 10^−4^ can be used [11].

### 6.3. Extrapolation to Galileo Full Operational Capability

As discussed above, the root cause of each one of the different events experienced by Galileo satellites has been investigated in detail by the Galileo Project Office to identify whether it could continue to occur when Galileo reaches the future FOC or will be eliminated thanks to the system configuration updates during this consolidation process. Table 10 provides the list of events that have been identified as representative of the FOC [5].

From the extrapolation to FOC, it follows that the two satellite events listed in Table 10, having been observed over a total of 5.28 × 10^5^ valid hours on the 43 months of data (see Table 9), imply an average Fault Rate of 4.7 × 10^−6^/sat/h.

Since the averaged fault duration resulting from the exposure time in Table 10 is only 42.5 min, it will take, again, 1 h for the MTTN to calculate $P_{sat}$. It should be noted that such an averaged duration value is very close to the 45 min considered by ICAO NSP [10] as the MTTN value that is expected to be achieved for the Narrow Faults in the future configuration of the Galileo system.

Taking MTTN equal to 1 h, it results in a $P_{sat}$ value of 4.7 × 10^−6^/sat. This value, estimated for the whole analyzed period, i.e., from 1 January 2017 to 31 July 2020, is much smaller than the value $P_{sat}$ = 7.8 × 10^−5^/sat given in Table 9 for the same time interval, and quite similar to the $P_{sat}$ = 5.3 × 10^−6^/sat value found when excluding the first six-month period.

## 7. Conclusions

A preliminary characterization of Galileo F/NAV broadcast orbit and clock errors has been made in this work based on more than three years of data since the Galileo Initial Service Open Service declaration, from 1 January 2017 to 31 July 2020. Results for GPS LNAV broadcast messages on the same data period and over the last 10 years, from 1 January 2010 to 31 July 2020, have also been determined to compare performances with the fully deployed and consolidated GPS constellation.

The methodology used in this study is based on the Stanford works [11,12], which involve complex algorithms for data cleansing and a procedure for anomaly detection and verification. This methodology has been directly implemented and applied to GPS and extended to Galileo data.

The observed orbit and clock errors in Galileo satellites are more tightly distributed than in the GPS, mainly for the along-track and cross-track errors. Events exceeding the 4.42 × SISA threshold have been identified, and their impact over the CDF was analyzed. It is worth mentioning that most of the detected events have been identified as unrepresentative of the future Galileo Full Operational Capability, and many of them were experienced during the first six-month period after the Galileo IS OS. When excluding this six-month period, the aggregated 1-CDF, incorporating all satellites, is well bounded beyond the probability level 1 × 10^−5^ by a Gaussian distribution with σ=4.

The observed nominal accuracy of Galileo satellites has been also characterized over the 43-month analyzed period and compared with the GPS determinations for the same period of time and over a longer period of more than 10 years. Results show smaller 68th and 95th Galileo percentiles for the along-track and cross-track errors than those in the GPS. Similar percentiles as in the GPS are found for the radial component and IURE, although a bias of several centimeters appears. The Galileo clock performs slightly better than the GPS clock, with smaller percentiles, but some global bias could have been absorbed by the clock alignment procedure applied to align the IGS time to the Galileo system time.

Finally, the NTE = 39.78 m threshold from Galileo commitments has been used to detect the satellite faults and to estimate the observed probability $P_{sat}$. When excluding the first six-month period of Galileo IS OS, the analysis over the last three-year window, from 1 August 2017 to 31 July 2020, shows very promising results. Only one satellite fault is found, the IOV E101 on 29 October 2019, lasting for 30 min. This single fault over this three-year period results in a fault probability $P_{sat}$ = 3.2 × 10^−6^/sat, which is far below the 1 × 10^−5^/sat commitment. Moreover, $P_{sat}$ has been also estimated using the NTE = 25.04 m threshold, from ICAO NSP of April 2020. In this case, the satellite fault experienced by the IOV satellite E101 on 26 December is included in the statistics, which leads to $P_{sat}$ = 5.3 × 10^−6^/sat when considering the last three-year time window, being, again, a very good result. The study ends with the extrapolation to the Galileo FOC, where only two events are thought to be representative of this future configuration. In this case, a value of $P_{sat}$ = 4.7 × 10^−6^/sat is estimated over the whole period of 43 months, i.e., from 1 January 2017 to 31 July 2020, which, again, broadly meets the 3 × 10^−5^/sat requirement.

It is worth noting that the Galileo system is still under the deployment phase, and this study is based on only about three and a half years of data. The results do not necessarily reflect the expected performance of the Galileo system once it is fully deployed. Thus, further studies should be performed in the future with larger historical data records to consolidate results.

## Figures and Tables

**Table 1:** Galileo F/NAV, from 1 January 2017 to 31 July 2020. Summary of events exceeding the NTE = 4.42 × SISA threshold. Events detected with the Space Approach are on the left side of the table, and verification results with the Ground Approach are on the right side. The “Duration” column indicates the elapsed time having WC URE over the 4.42 × SISA threshold.

Potential Anomalies Found with Space Approach (NTE = 4.42 × SISA)										Potential Anomalies Found with Ground Approach								Final Decision
YYDOY	SVN	PRN	Start Time		Duration (min)	Anomaly		WC URE (m)	SISA (m)	Start Time	Duration (min)	WC URE (m)	Ref. Station	Num. of Stations that Decide			Algorithm Decision	
						Type	Value (m)							Anom.	Nominal	Untrack		
17066	E206	30	7 March 2017	03:15	40	clock	340.9	341.9	3.12	03:21	38.5	341.9	SEYG	11	0	39	TRUE	TRUE
17134	E101	11	14 May 2017	13:30	60	clock	14.9	15.0	3.12	11:42	287.5	17.6	WGTN	11	1	39	PARADOX	TRUE
17134	E102	12	14 May 2017	12:20	25	clock	14.4	14.4	3.12	10:25	144.5	14.4	WGTN	13	0	37	TRUE	TRUE
17134	E205	24	14 May 2017	17:35	395	clock	20.3	20.3	3.12	15:38	309.5	16.9	YEL2	24	3	23	PARADOX	TRUE
17134	E206	30	14 May 2017	13:30	625	clock	26.6	26.9	3.12	20:17	222.0	26.9	YEL2	22	0	28	TRUE	TRUE
17134	E208	08	14 May 2017	23:35	25	clock	13.8	14.2	3.12	23:45	9.5	14.1	RGDG	1	7	42	PARADOX	TRUE
17134	E211	02	14 May 2017	11:55	30	clock	14.6	14.9	3.12	08:59	150.5	14.3	MAYG	9	6	35	PARADOX	TRUE
17135	E205	24	15 May 2017	00:00	70	clock	20.6	20.6	3.12	00:03	66.5	20.6	RGDG	11	1	38	PARADOX	TRUE
17135	E206	30	15 May 2017	00:00	190	clock	31.4	36.7	3.12	00:03	157.0	33.3	YEL2	9	0	41	TRUE	TRUE
17135	E208	08	15 May 2017	00:00	105	clock	15.1	15.1	3.12	01:02	47.5	15.1	RGDG	5	7	38	PARADOX	TRUE
17157	E203	26	6 June 2017	05:50	1085	clock	491.3	491.9	3.12	12:27	163.5	491.9	YEL2	31	0	19	TRUE	TRUE
17158	E203	26	7 June 2017	00:00	430	clock	460.4	472.8	3.12	01:09	360.5	472.8	YEL2	21	0	29	TRUE	TRUE
17332	E205	24	28 November 2017	06:45	185	clock	16.2	16.6	3.12	07:27	144.5	16.6	YEL2	17	1	32	PARADOX	TRUE
17360	E101	11	26 December 2017	07:45	385	clock	27.2	27.2	3.12	05.48	140.5	14.7	VIGO	28	7	15	PARADOX	TRUE
18248	E206	30	5 September 2018	02:20	10	eph.	18.8	17.8	3.12	02:08	21.5	17.8	TLSE	20	7	23	PARADOX	TRUE
19066	E103	19	7 March 2019	12:15	125	eph.	22.2	18.8	3.12	14:03	17.5	18.8	KIRU	8	0	42	TRUE	TRUE
19302	E101	11	29 October 2019	18.10	30	clock	431.9	432.1	3.12	18:08	36.0	432.1	STHL	18	0	32	TRUE	TRUE

**Table 2:** GPS LNAV, from 1 January 2010 to 31 July 2020. Summary of events detected based on NTE = 4.42 × IAURA. The same content as Table 1 for the Space Approach. The Ground Approach has also confirmed all these events.

Events found with Space Approach (NTE = 4.42 × IAURA)									
YYDOY	SVN	PRN	Start Time		Duration (min)	Anomaly		WC URE (m)	IAURA (m)
						Type	Value (m)		
10053	G030	30	22 February 2010	20:55	20	clock	42.8	42.9	3.40
10115	G039	09	25 April 2010	19:45	10	eph.	44.9	11.3	2.40
12169	G059	19	17 June 2012	00:15	20	eph.	1899.0	451.5	2.40

**Table 3:** Galileo F/NAV Nominal Accuracy, from 1 January 2017 to 31 July 2020.

		From 1 January 2017 to 31 July 2020: gAGE Consolidated Broadcast + MGEX Precise Orbits and Clocks																									
		Radial (cm)				Along-Track (cm)				Cross-Track (cm)				Clock (cm)				WC URE (cm)				N. Samples	IURE (cm) (Acum. Dodec.)				
	SVN	$\bar{x}$	68th	95th	σ	$\bar{x}$	68th	95th	σ	$\bar{x}$	68th	95th	σ	$\bar{x}$	68th	95th	σ	$\bar{x}$	68th	95th	σ		$\bar{x}$	68th	95th	σ	N. Sampl.
IOV	E101	5.1	13	28	15	−5.5	28	59	32	−0.1	18	35	18	−5.3	17	38	25	15.6	24	44	27	353,796	10.3	17	35	24	2,446,525
IOV	E102	8.6	16	32	15	−9.6	30	65	32	0.3	19	37	19	−17.1	25	46	26	33.3	38	56	26	366,125	25.4	31	48	24	2,568,002
IOV	E103	3.1	12	25	15	−6.8	33	70	52	1.8	20	40	21	−9.3	18	38	18	19.0	27	46	25	353,354	12.5	18	37	17	2,462,407
FOC	E203	13.7	20	40	18	−7.1	26	57	34	0.0	17	34	17	−2.1	16	35	21	20.6	28	50	24	360,845	15.5	22	43	20	2,533,431
FOC	E204	7.0	14	30	28	−6.5	28	64	35	−0.5	17	35	28	11.2	20	43	27	−5.2	21	45	33	97,644	−4.4	15	36	26	679,576
FOC	E205	10.3	15	31	14	−3.3	24	53	28	1.6	16	32	16	4.0	13	30	24	8.7	17	33	28	365,578	6.2	12	27	24	2,542,545
FOC	E206	9.4	15	30	14	−4.2	25	54	29	0.6	16	33	17	5.8	14	32	25	5.6	17	33	28	364,742	3.6	11	26	24	2,526,134
FOC	E207	9.3	14	28	13	−7.5	25	52	26	−0.5	16	32	16	10.1	17	36	15	−0.9	16	31	19	326,371	−0.9	11	24	14	2,293,869
FOC	E208	11.7	17	34	15	−6.9	25	54	35	0.6	15	32	16	−1.1	13	30	27	17.0	24	42	30	364,725	12.6	18	36	26	2,532,747
FOC	E209	11.3	17	33	14	−2.5	24	54	30	1.0	17	33	17	1.0	11	28	14	13.9	22	40	19	363,586	10.3	16	34	15	2,556,984
FOC	E210	10.2	15	31	14	−6.0	25	54	28	1.8	16	33	17	2.6	16	36	18	10.1	19	44	22	362,894	7.4	13	37	18	2,522,405
FOC	E211	11.6	17	34	16	−2.2	24	52	28	1.4	16	32	16	−0.8	13	31	25	16.1	23	44	30	362,281	12.3	17	38	26	2,517,599
FOC	E212	11.0	17	36	15	−3.8	24	54	28	−0.1	16	32	16	−1.9	12	28	14	17.3	23	43	19	307,404	12.7	17	37	16	2,160,517
FOC	E213	11.6	17	37	17	−4.5	24	53	33	1.3	16	33	17	−1.0	13	29	15	17.0	23	43	21	306,383	12.5	17	37	17	2,137,544
FOC	E214	13.4	20	41	17	−3.2	24	52	32	1.4	17	33	17	−5.9	14	30	16	24.5	30	52	21	323,637	19.2	24	46	18	2,255,881
FOC	E215	5.6	11	24	13	−1.5	23	49	26	1.3	17	33	17	4.3	13	29	16	2.5	15	27	19	181,434	1.2	9	20	14	1,258,327
FOC	E216	5.8	11	24	12	−2.7	22	49	26	0.9	15	31	16	1.6	12	28	14	6.6	15	28	16	199,853	4.1	10	21	12	1,385,543
FOC	E217	5.3	11	22	11	−3.0	24	49	26	−0.3	16	33	17	4.6	13	28	15	1.9	14	26	18	202,319	0.7	9	19	14	1,407,647
FOC	E218	6.0	11	24	13	−4.6	23	48	32	0.8	16	31	16	3.2	13	30	16	4.9	15	28	18	199,516	2.8	9	22	12	1,382,738
FOC	E219	6.0	13	26	13	−2.0	25	53	29	−0.3	17	33	17	6.1	17	33	16	0.7	16	28	17	147,133	−0.1	9	21	11	1,029,590
FOC	E220	5.3	12	25	13	−5.2	24	50	30	−0.5	18	33	17	2.8	15	31	15	4.7	16	28	17	144,999	2.5	9	21	11	1,000,198
FOC	E221	5.6	13	25	12	−4.6	24	52	26	0.0	18	33	17	3.8	15	32	15	3.7	16	28	16	145,985	1.7	10	21	11	1,022,116
FOC	E222	4.8	13	26	16	−2.1	25	54	52	−1.0	17	33	19	1.6	15	32	20	5.9	17	31	25	144,747	3.1	10	23	14	998,716
**ALL**	**IOV**	**5.6**	**14**	**29**	**15**	**−7.3**	**30**	**65**	**40**	**0.7**	**19**	**38**	**19**	**−10.6**	**20**	**41**	**24**	**22.7**	**31**	**51**	**27**	**1,073,275**	**16.2**	**23**	**42**	**23**	**7,476,934**
**ALL**	**FOC**	**9.7**	**15**	**32**	**15**	**−4.3**	**24**	**53**	**31**	**0.6**	**16**	**33**	**17**	**1.8**	**14**	**32**	**20**	**10.7**	**20**	**41**	**24**	**5,272,076**	**7.7**	**14**	**34**	**20**	**36,744,107**
**ALL**	**ALL**	**9.0**	**15**	**32**	**15**	**−4.8**	**25**	**55**	**32**	**0.6**	**17**	**34**	**17**	**−0.3**	**15**	**34**	**21**	**12.7**	**22**	**44**	**25**	**6,345,351**	**9.2**	**15**	**36**	**21**	**44,221,041**

**Table 4:** GPS LNAV Nominal Accuracy, from 1 January 2017 to 31 July 2020.

		From 1 January 2017 to 31 July 2020: gAGE Consolidated Broadcast + MGEX Precise Orbits and Clocks																									
		Radial (cm)				Along-Track (cm)				Cross-Track (cm)				Clock (cm)				WC URE (cm)				N. Samples	IURE (cm) (Acum. Dodec.)				
	SVN	$\bar{x}$	68th	95th	σ	$\bar{x}$	68th	95th	σ	$\bar{x}$	68th	95th	σ	$\bar{x}$	68th	95th	σ	$\bar{x}$	68th	95th	σ		$\bar{x}$	68th	95th	σ	N. Sampl.
IIA	G034	−0.6	18	36	18	6.4	96	225	110	0.3	53	98	51	−6.1	62	155	72	8.2	87	187	93	184,456	3.3	63	158	74	1,300,383
IIR	G041	−1.1	11	24	12	49.3	106	226	102	0.2	41	81	41	−7.7	28	64	31	11.7	54	100	54	360,479	7.0	32	71	35	2,639,671
IIR	G043	−0.1	10	21	11	18.9	86	192	92	−0.4	36	72	37	−3.2	36	88	43	3.8	59	115	62	365,426	3.0	39	93	45	2,454,557
IIR	G044	1.4	13	27	13	−9.6	85	188	92	0.4	38	78	39	−5.2	82	218	97	9.0	104	244	115	365,428	6.6	83	219	98	2,400,351
IIR	G045	0.5	14	30	15	12.7	104	226	112	1.5	54	107	54	−3.3	26	59	30	6.9	56	99	56	365,310	5.3	33	72	36	2,399,917
IIR	G046	2.8	14	29	14	−52.1	118	267	120	−0.9	47	93	47	−2.2	37	95	45	8.1	69	136	71	365,141	6.6	43	105	50	2,472,920
IIR	G047	1.9	13	26	14	−5.8	92	196	101	0.4	33	64	33	−5.1	23	48	23	12.3	45	78	44	365,233	7.5	27	57	28	2,473,181
IIR	G048	0.9	13	26	13	−4.7	88	193	94	0.9	54	105	53	2.6	38	87	42	−3.9	62	118	63	363,994	−1.8	41	92	45	2,324,785
IIR	G050	1.7	13	26	13	2.7	79	170	85	0.1	34	67	34	4.4	26	54	27	−3.3	45	78	44	364,960	−3.0	27	59	29	2,376,931
IIR	G051	0.8	10	22	11	2.3	84	180	90	0.0	48	105	51	0.2	23	49	24	1.5	46	80	46	365,291	2.0	27	58	29	2,468,535
IIR	G052	1.2	12	24	12	0.0	83	183	91	−0.1	38	81	40	−5.0	38	91	43	8.5	60	118	62	364,962	5.7	40	95	45	2,343,561
IIR	G053	0.1	14	28	14	18.4	117	267	128	0.4	35	72	36	1.4	52	145	66	−1.3	79	180	89	365,110	−1.5	53	146	67	2,390,098
IIR	G054	1.1	12	24	12	−13.5	92	197	99	1.3	54	97	51	−4.2	31	86	41	6.9	56	114	61	110,854	6.4	35	90	43	749,849
IIR	G055	0.1	12	24	12	9.9	95	212	103	0.1	34	71	36	2.5	21	43	22	−4.7	44	78	43	364,942	−2.2	25	53	26	2,395,559
IIR	G056	1.2	13	26	13	−15.9	77	163	80	−0.3	35	71	36	−2.3	22	46	23	6.3	42	72	41	365,333	3.8	25	53	26	2,438,184
IIR	G057	1.6	15	30	15	7.8	120	263	130	−0.1	50	95	49	3.9	40	111	54	−8.3	70	152	78	364,843	−3.4	43	118	57	2,312,041
IIR	G058	1.0	15	28	15	11.6	87	189	93	−0.5	39	77	39	−5.1	24	49	25	11.0	46	80	45	365,127	6.4	28	58	29	2,448,912
IIR	G059	1.1	11	24	12	10.2	85	182	90	−0.7	45	83	43	2.7	23	50	24	−2.6	46	80	45	365,432	0.0	27	58	29	2,649,750
IIR	G060	2.1	12	24	12	−19.9	90	192	93	0.7	47	87	46	0.2	22	47	23	3.1	47	80	46	330,981	1.4	27	58	29	2,201,256
IIR	G061	−0.5	12	26	13	31.8	102	230	108	0.9	40	79	40	−2.2	24	55	27	3.1	52	94	52	365,266	2.5	30	65	32	2,418,084
IIF	G062	−0.9	21	42	21	23.1	101	221	110	−0.2	43	83	42	5.0	27	53	26	−8.9	57	101	57	365,034	−4.8	37	76	38	2,626,341
IIF	G063	1.5	18	38	19	−21.3	106	232	113	−0.6	50	90	47	5.8	36	79	41	−6.7	66	119	66	363,301	−5.4	44	95	48	2,385,276
IIF	G064	0.4	16	32	16	16.4	82	182	89	0.1	32	65	33	5.8	25	51	25	−7.7	50	87	49	365,008	−5.3	32	67	33	2,448,277
IIF	G065	0.0	25	51	26	19.5	103	238	117	−0.6	33	67	33	0.8	120	243	121	1.2	148	275	145	365,176	−1.3	123	248	124	2,446,742
IIF	G066	0.4	16	31	16	−13.8	81	176	88	−0.4	39	77	39	1.8	22	43	22	−1.6	49	82	47	364,855	−0.8	31	62	32	2,457,384
IIF	G067	0.5	19	37	19	7.2	103	233	113	−0.1	33	71	35	7.3	26	55	26	−9.6	56	99	55	365,087	−6.3	36	73	36	2,324,301
IIF	G068	1.0	15	31	15	6.5	75	173	86	0.6	32	65	33	1.1	24	54	29	0.3	46	87	49	365,082	0.6	29	66	34	2,373,489
IIF	G069	0.5	18	37	19	0.6	93	204	104	−0.3	38	76	39	−10.2	41	85	42	15.3	67	115	64	365,002	13.3	46	95	47	2,425,257
IIF	G070	0.7	13	28	14	−3.7	88	202	100	0.5	36	72	36	−2.3	19	41	22	5.3	45	82	46	365,017	3.1	27	59	30	2,568,890
IIF	G071	−0.5	19	37	19	9.9	94	210	107	0.2	42	83	42	−0.5	26	55	27	0.5	56	98	56	364,879	0.7	36	75	38	2,338,517
IIF	G072	1.3	22	45	22	−30.8	112	244	118	−0.2	47	89	47	−0.2	96	207	101	2.3	126	242	126	364,969	2.4	99	213	104	2,312,670
IIF	G073	0.2	14	29	14	−5.4	92	203	103	0.6	48	92	47	−8.1	24	54	25	13.2	52	90	50	365,041	9.4	32	68	32	2,463,582
**IIA**	**ALL**	**−0.6**	**18**	**36**	**18**	**6.4**	**96**	**225**	**110**	**0.3**	**53**	**98**	**51**	**−6.1**	**62**	**155**	**72**	**8.2**	**87**	**187**	**93**	**184,456**	**3.3**	**63**	**158**	**74**	**1,300,383**
**IIR**	**ALL**	**0.9**	**13**	**26**	**13**	**3.6**	**93**	**209**	**103**	**0.2**	**41**	**85**	**43**	**−1.3**	**29**	**83**	**42**	**3.5**	**54**	**114**	**62**	**6,644,112**	**2.7**	**33**	**88**	**45**	**44,358,142**
**IIF**	**ALL**	**0.4**	**18**	**38**	**19**	**0.7**	**94**	**212**	**106**	**0.0**	**39**	**79**	**40**	**0.5**	**32**	**118**	**53**	**0.3**	**61**	**150**	**75**	**4,378,451**	**0.5**	**41**	**122**	**58**	**29,170,726**
**ALL**	**ALL**	**0.7**	**14**	**31**	**16**	**2.5**	**94**	**210**	**104**	**0.1**	**41**	**83**	**42**	**−0.7**	**31**	**97**	**47**	**2.3**	**56**	**130**	**68**	**11,207,019**	**1.8**	**36**	**103**	**51**	**74,829,251**

**Table 5:** GPS LNAV Nominal Accuracy, from 1 January 2010 to 31 July 2020.

		From 1 January 2010 to 31 July 2020: gAGE Consolidated Broadcast + NGA Precise Orbits and Clocks																									
		Radial (cm)				Along-Track (cm)				Cross-Track (cm)				Clock (cm)				WC URE (cm)				N. Samples	IURE (cm) (Acum. Dodec.)				
	SVN	$\bar{x}$	68th	95th	σ	$\bar{x}$	68th	95th	σ	$\bar{x}$	68th	95th	σ	$\bar{x}$	68th	95th	σ	$\bar{x}$	68th	95th	σ		$\bar{x}$	68th	95th	σ	N. Samples
IIA	G023	−2.2	25	46	24	19.8	100	230	112	0.1	42	86	44	−2.1	61	131	65	1.8	80	156	82	634,169	1.1	55	128	62	4,080,462
IIA	G024	−0.5	42	77	41	−11.1	152	329	170	0.5	41	84	42	−1.3	141	280	142	5.0	169	305	165	182,285	1.1	132	265	133	1,230,973
IIA	G026	−2.5	15	35	18	14.2	103	252	123	−0.7	38	77	39	3.8	47	117	57	−8.0	75	156	81	521,082	−6.0	52	125	61	3,485,740
IIA	G027	−1.4	83	126	74	15.2	251	473	247	−1.5	46	88	45	7.0	197	355	188	−0.9	220	365	208	252,287	−11.0	158	301	155	1,819,735
IIA	G030	−2.5	24	50	25	26.3	114	269	130	−0.3	33	67	34	6.5	134	259	133	−8.4	161	290	156	141,581	−9.7	133	259	132	986,392
IIA	G033	−1.8	26	51	26	−1.0	120	257	129	−0.7	41	81	41	−1.4	129	255	129	1.4	154	283	151	480,756	0.3	126	253	127	3,229,855
IIA	G034	−1.8	17	35	18	6.1	101	228	113	0.0	39	79	40	0.5	68	159	76	−1.8	91	190	97	796,131	−2.7	68	161	77	5,337,739
IIA	G035	−0.8	22	49	25	6.5	88	196	99	−0.3	47	91	46	−3.0	146	308	156	4.8	171	335	175	161,036	2.5	147	309	156	1,053,649
IIA	G036	−1.9	16	35	18	6.1	111	248	123	−0.4	41	81	41	−8.7	75	187	86	9.2	102	219	109	433,611	6.4	77	189	88	2,905,140
IIA	G038	−1.8	29	56	29	−8.1	128	276	140	1.4	81	138	75	−0.7	135	265	134	0.6	165	297	160	505,126	−0.6	132	262	132	3,305,906
IIA	G039	−2.5	42	73	40	22.8	155	312	158	−0.2	34	69	36	2.6	141	273	139	−2.7	168	298	162	453,215	−5.6	132	260	132	2,996,678
IIA	G040	−1.7	20	41	21	−4.1	105	237	121	−0.8	38	76	38	5.3	123	245	123	−7.3	148	276	146	579,819	−7.0	124	249	125	3,866,837
IIR	G041	−0.5	12	24	12	10.5	96	210	104	0.3	43	85	43	−3.7	28	65	33	5.5	53	99	55	1,093,820	4.1	32	72	36	7,527,301
IIR	G043	0.5	11	23	12	−7.0	86	190	95	−0.4	42	83	42	−1.9	35	83	41	3.3	56	112	60	1,098,316	3.0	38	89	44	7,174,694
IIR	G044	0.8	13	27	13	−16.3	91	199	97	0.3	42	84	42	−3.3	94	224	104	6.0	117	250	123	1,099,057	4.5	95	225	105	7,283,867
IIR	G045	0.1	14	28	14	3.1	102	224	112	1.3	54	105	54	−2.3	27	60	31	4.9	55	99	56	1,098,722	3.8	32	71	36	7,176,231
IIR	G046	0.1	14	28	14	−21.1	108	239	117	−1.1	44	88	44	0.1	43	116	55	−0.5	71	151	78	1,097,737	1.3	47	122	58	7,480,736
IIR	G047	0.8	13	26	13	−5.2	93	204	102	0.6	49	103	51	3.5	40	139	63	−3.9	65	169	82	1,097,713	−1.9	43	140	64	7,427,244
IIR	G048	0.3	13	27	14	−3.2	84	186	92	0.8	55	103	54	0.5	36	83	40	−0.9	59	112	61	1,097,593	0.4	38	88	43	6,982,467
IIR	G050	0.2	13	25	13	−1.3	80	168	86	0.0	34	68	35	1.2	25	52	26	−0.9	44	77	44	1,098,283	−1.4	27	57	28	7,163,104
IIR	G051	0.0	11	23	12	−10.9	89	195	97	−0.2	48	96	49	−0.5	23	51	26	1.2	48	84	48	1,097,335	1.8	28	60	30	7,391,918
IIR	G052	0.6	12	25	12	0.0	90	198	98	−0.2	41	83	42	−2.5	42	105	50	4.2	65	134	70	1,098,270	3.0	44	109	52	7,038,834
IIR	G053	0.0	13	28	14	2.6	114	254	126	0.6	51	105	53	1.9	49	142	65	−1.4	78	179	89	1,098,471	−1.6	52	144	66	7,290,358
IIR	G054	−0.3	13	26	14	6.6	93	204	101	1.5	50	96	50	−2.9	26	70	36	3.6	51	103	57	844,431	3.5	30	76	39	5,909,974
IIR	G055	−0.3	12	25	13	−3.6	94	203	101	−0.1	40	81	41	1.3	21	44	22	−2.5	45	77	44	1,098,372	−1.2	25	53	26	7,146,200
IIR	G056	−0.3	13	25	13	−8.1	81	174	87	−0.3	42	83	43	−2.0	22	46	23	3.5	43	74	42	1,098,440	2.8	25	54	27	7,302,290
IIR	G057	0.2	15	29	15	14.8	118	265	130	−0.2	46	93	47	3.3	41	115	56	−9.1	71	156	80	1,098,204	−2.6	44	122	59	6,966,483
IIR	G058	0.2	15	28	15	9.3	97	212	106	−0.3	38	76	39	−2.8	24	50	26	6.1	48	84	48	1,098,471	3.6	28	60	31	7,377,606
IIR	G059	0.0	11	23	11	−4.1	83	179	90	−0.8	47	89	46	−0.4	24	54	26	0.8	47	83	47	1,098,801	1.8	28	62	30	7,516,062
IIR	G060	1.2	12	24	12	−10.5	85	181	89	0.5	45	89	45	0.2	22	46	23	1.8	45	77	44	1,064,220	0.6	26	55	27	7,109,733
IIR	G061	−0.6	11	23	11	15.4	85	194	94	0.8	43	83	43	−2.0	25	56	30	2.6	49	88	50	1,098,004	2.4	29	64	33	7,292,988
IIF	G062	−0.5	18	36	18	5.6	92	201	103	−0.3	38	75	38	0.7	21	46	23	−1.5	51	90	51	1,029,880	−0.5	31	67	33	7,452,310
IIF	G063	0.9	16	33	16	−25.8	102	223	108	0.2	45	84	44	0.5	25	64	32	1.0	55	105	58	909,943	0.5	34	80	40	5,988,329
IIF	G064	0.0	16	32	17	21.9	81	183	92	0.1	34	70	35	1.8	23	50	27	−2.4	49	89	51	636,049	−1.9	31	67	35	4,251,589
IIF	G065	−0.2	23	47	24	8.9	105	236	117	−0.2	36	71	36	0.3	112	234	115	0.9	140	267	139	798,327	−1.2	115	240	118	5,375,499
IIF	G066	0.4	16	31	16	−11.9	87	189	98	−0.3	35	71	36	1.1	20	42	21	−0.7	48	83	48	734,798	−0.3	30	62	32	4,952,788
IIF	G067	0.6	17	36	18	0.1	94	216	107	−0.1	37	76	38	3.3	23	50	24	−3.5	52	94	52	633,047	−1.8	32	69	34	4,037,496
IIF	G068	0.6	15	33	16	7.3	80	182	91	0.5	34	68	35	−1.0	22	52	27	2.8	47	89	50	604,580	2.2	30	66	34	3,923,233
IIF	G069	0.8	17	37	19	−4.0	94	214	110	−0.1	37	75	38	−6.5	32	78	37	10.8	61	112	62	579,700	9.2	40	90	44	3,850,647
IIF	G070	0.5	14	33	17	−2.9	92	215	108	0.5	36	74	37	−2.4	21	47	25	5.2	48	93	52	449,579	3.2	29	66	35	3,162,919
IIF	G071	−0.7	19	38	19	10.5	96	213	108	0.4	39	79	40	−0.7	24	53	26	0.5	56	97	56	542,495	0.4	35	74	38	3,473,748
IIF	G072	1.2	22	44	22	−34.2	112	246	119	−0.1	46	87	46	0.5	93	201	98	1.3	123	238	124	509,746	1.5	96	209	101	3,231,799
IIF	G073	0.2	14	31	16	−5.3	90	204	105	0.5	50	95	49	−9.4	24	56	25	15.3	53	95	52	475,901	10.6	33	71	34	3,211,411
**IIA**	**ALL**	**−1.9**	**24**	**61**	**30**	**7.3**	**118**	**277**	**136**	**−0.2**	**42**	**91**	**45**	**0.5**	**99**	**242**	**113**	**−0.9**	**126**	**269**	**134**	**5,141,098**	**−2.4**	**96**	**234**	**110**	**34,299,106**
**IIR**	**ALL**	**0.2**	**13**	**26**	**13**	**−1.6**	**93**	**206**	**102**	**0.1**	**45**	**90**	**46**	**−0.6**	**31**	**93**	**46**	**1.2**	**55**	**124**	**65**	**20,574,260**	**1.5**	**35**	**97**	**48**	**136,558,090**
**IIF**	**ALL**	**0.3**	**17**	**37**	**19**	**−2.4**	**94**	**211**	**107**	**0.0**	**39**	**78**	**39**	**−0.6**	**29**	**113**	**51**	**1.8**	**59**	**148**	**73**	**7,904,045**	**1.3**	**38**	**120**	**56**	**52,911,768**
**ALL**	**ALL**	**−0.1**	**15**	**35**	**18**	**−0.4**	**96**	**219**	**109**	**0.1**	**43**	**87**	**44**	**−0.4**	**36**	**141**	**62**	**1.1**	**63**	**172**	**81**	**33,619,403**	**0.8**	**41**	**141**	**63**	**223,768,964**

**Table 6:** The same as Table 1 for the Space Approach, but considering the NTE = 39.78 m threshold. The Ground Approach has also confirmed all these events.

Events Found with Space Approach Using NTE = 39.78 m									
YYDOY	SVN	PRN	Start Time		Duration (min)	Anomaly		WC URE (m)	SISA (m)
						Type	Value (m)		
17066	E206	30	07 March 2017	03:15	40	clock	340.9	341.9	3.12
17157	E203	26	06 June 2017	05:50	1085	clock	491.3	491.9	3.12
17158			07 June 2017	00:00	430	clock	460.4	472.8	3.12
19302	E101	11	29 October 2019	18.10	30	clock	431.9	432.1	3.12

**Table 7:** Summary of faults detected using NTE *=* 39.78 m.

Year	Number Satellite Fault Events			Faults Duration (h)			Total Signal Valid Hours
	IOV	FOC	Total	IOV	FOC	Total	
1 August 2017–31 December 2017	0	0	0	0	0	0	0.54 × 10^5^
Full 2017	0	2	2	0	25.9	25.9	1.12 × 10^5^
Full 2018	0	0	0	0	0	0	1.34 × 10^5^
Full 2019	1	0	1	0.5	0	0.5	1.76 × 10^5^
1 January 2020–31 July 2020	0	0	0	0	0	0	1.07 × 10^5^

**Table 8:** Fault Rate and Probability of Galileo Satellite Fault, estimated with NTE *=* 39.78 m.

Years	Total Satellite Fault Events			Total Valid (h)	Estimated Mean Fault Rate	Average Fault Duration	MTTN (h)	*Psat* (/sat)
	IOV	FOC	Total		(/sat/h)	(h)		
1 January 2017–31 July 2020	1	2	3	5.28 × 10^5^	6.6 × 10^−6^	8.81	8.81	5.8 × 10^−5^
1 August 2017–31 July 2020	1	0	1	4.70 × 10^5^	3.2 × 10^−6^	0.50	1.00	3.2 × 10^−6^

**Table 9:** Fault Rate and Probability of Galileo Satellite Fault, estimated with NTE = 25.04 m.

Years	Total Satellite Fault Events			Total Valid (h)	Estimated Mean Fault Rate	Average Fault Duration	MTTN (h)	*Psat* (/sat)
	IOV	FOC	Total		(/sat/h)	(h)		
1 January 2017–31 July 2020	2	2	4	5.28 × 10^5^	8.5 × 10^−6^	9.13	9.13	7.8 × 10^−5^
1 August 2017–31 July 2020	2	0	2	4.70 × 10^5^	5.3 × 10^−6^	0.42	1.00	5.3 × 10^−6^

**Table 10:** List of retained events after FOC extrapolation and associated exposure time (also extrapolated) between 1 January 2017 and 31 July 2020.

Date	SVN	PRN	Extrapolated to FOC	
			Exposure Time (min)	WC URE (m)
6 June 2017	E203	26	50	>40
29 October 2019	E101	11	35	>40

## References

[1] European GNSS Service Centre (GSA). https://www.gsc-europa.eu/system-service-status/constellation-information.

[2] GNSS (2010). Phase II of the GNSS Evolutionary Architecture Study. http://www.faa.gov/about/office_org/headquarters_offices/ato/service_units/techops/navservices/gnss/library/documents/media/GEASPhaseII_Final.pdf..

[3] Blanch J., Walker T., Enge P.K., Lee Y., Pervan B., Rippl M., Spletter A., Kropp V. (2015). Baseline advanced RAIM user algorithm and possible improvements. IEEE Trans. Aerosp. Electron. Syst..

[4] Walter T., Joerger M., Pervan B. Determination of fault probabilities for ARAIM. Proceedings of the 2016 IEEE/ION Position, Location and Navigation Symposium (PLANS).

[5] Galluzo G., Wallner S., Pericacho J.G., Criado O., García C., Sobrero F.J., Brieden P., Binder K., Battista G., Odriozola M. Prototyping of galileo URA determination with TGVF and extended galileo performance characterisation for SoL applications. Proceedings of the 33rd International Technical Meeting of the Satellite Division of The Institute of Navigation (ION GNSS+ 2020).

[6] Walter T., Blanch J., Choi M.J., Reid T., Enge P. Incorporating GLONASS into aviation RAIM receivers. Proceedings of the ION International Technical Meeting.

[7] Working Group C-ARAIM Technical Subgroup (2016). EU-U.S.. Cooperation on Satellite Navigation.

[8] United States Department of Defense Global Positioning System Standard Positioning Service Performance Standard. https://www.gps.gov/technical/ps/2020-SPS-performance-standard.pdf.

[9] (2019). European GNSS (Galileo) Open Service—Service Definition Document, Issue 1.1. https://www.gsc-europa.eu/sites/default/files/sites/all/files/Galileo-OS-SDD_v1.1.pdf.

[10] Chatre E., Boyero J.P., Martini I., Sgammini M., Wallner S., Cosson F., Brieden P., Laura D., Canestri D. Galileo performance characterization for horizontal ARAIM. Proceedings of the ICAO Navigation System Panel (NSP).

[11] Walter T., Blanch J. KEYNOTE—Characterization of GNSS clock and ephemeris errors to support ARAIM. Proceedings of the ION 2015 Pacific PNT Meeting.

[12] Heng L. (2012). Safe Satellite Navigation with Multiple Constellations: Global Monitoring of GPS and GLONASS Signal-in-Space Anomalies. Ph.D. Dissertation.

[13] Guurter W. (2012). RINEX: The Receiver Independent Exchange Format.

[14] Global Positioning System Wing (GPSW) (2010). Interface Specification IS-GPS-200E. https://www.navcen.uscg.gov/pdf/gps/IS-GPS-200E_Final_08Jun10.pdf.

[15] Sanz J., Juan Zornoza J.M., Hernández-Pajares M. (2013). GNSS Data Processing, Volume 1: Fundamentals and Algorithms.

[16] Ibáñez D., Rovira-Garcia A., Alonso M.T., Sanz J., Juan J.M., González-Casado G., Lopez-Martínez M. (2020). EGNOS 1046 Maritime Service Assessment. Sensors.

[17] Montenbruck O., Steigenberger P., Prange L., Deng Z., Zhao Q., Perosanz F., Romero I., Noll C., Stürze A., Weber G. (2017). The Multi-GNSS Experiment (MGEX) of the International GNSS Service (IGS)—Achievements, prospects and challenges. Adv. Space Res..

[18] (2019). ANTEX Files Server. European GNSS Service Centre. https://www.gsc-europa.eu/sites/default/files/sites/all/files/GSAT_2023.atx.txt.

[19] National Geospatial Agency Server. ftp://ftp.nga.mil/pub2/gps/apcpe.

[20] (2016). European GNSS (Galileo) Open Service—Signal-In-Space Interface Control Document, Issue 1.3. https://www.gsc-europa.eu/sites/default/files/sites/all/files/Galileo-OS-SIS-ICD.pdf.

[21] NAGU. https://www.gsc-europa.eu/system-status/user-notifications-archive.

[22] Cedric R., Bonhoure B., Suard N., Mabilleau M., Vuillaume J., Dulery C., Lapeyre D., Sauce C. Galileo ephemeris consolidation and control analysis—GECCO. Proceedings of the 28th International Technical Meeting of the Satellite Division of The Institute of Navigation (ION GNSS+ 2015).

[23] Serenad Server from CNES. ftp://serenad-public.cnes.fr/SERENAD0/FROM_NTMFV2.

[24] Juan J.M., Sanz J., Rovira-Garcia A., González-Casado G., Ventura-Traveset J., Cacciapuoti L., Schoenemann E. A New Approach to Improve Satellite Clock Estimates, Removing the Inter-day Jumps. Proceedings of the 51st Annual Precise Time and Time Interval Systems and Applications Meeting.

[25] Walter T., Rife J., Blanch J. Treatment of Biased Error Distributions in SBAS. Proceedings of the 2004 International Symposium on GNSS/GPS.

[26] DeCleene B. Defining pseudorange integrity—Overbounding. Proceedings of the 13th International Technical Meeting of the Satellite Division of The Institute of Navigation (ION GPS 2000).

[27] Brieden P., Wallner S., Canestri E., Joly D., Sanz J., Martini I., Nuckelt A., Battista G., Lauria D., Luongo F. Galileo characterization as input to H-ARAIM and SBAS DFMC. Proceedings of the 32nd International Technical Meeting of the Satellite Division of The Institute of Navigation (ION GNSS+ 2019).

[28] Guo F., Li X., Zhang X., Wang J. (2017). Assessment of precise orbits and clock products for Galileo, Beidou and QZSS from IGS Multi-GNSS Experiment (MGEX). GPS Solut..

